# DDR1 promotes hepatocellular carcinoma metastasis through recruiting PSD4 to ARF6

**DOI:** 10.1038/s41388-022-02212-1

**Published:** 2022-02-09

**Authors:** Xiaochao Zhang, Yabing Hu, Yonglong Pan, Yixiao Xiong, Yuxin Zhang, Mengzhen Han, Keshuai Dong, Jia Song, Huifang Liang, Zeyang Ding, Xuewu Zhang, He Zhu, Qiumeng Liu, Xun Lu, Yongdong Feng, Xiaoping Chen, Zhanguo Zhang, Bixiang Zhang

**Affiliations:** 1 Hepatic Surgery Center, Tongji Hospital, Tongji Medical College, Huazhong University of Science and Technology, Wuhan, China; 2 Clinical Medical Research Center of Hepatic Surgery at Hubei Province, Wuhan, China; 3 Key Laboratory of Organ Transplantation, Ministry of Education, Wuhan, P. R. China; 4 Key Laboratory of Organ Transplantation, National Health Commission, Wuhan, P. R. China; 5 Key Laboratory of Organ Transplantation, Chinese Academy of Medical Sciences, Wuhan, China; 6 Dermatology, Tongji Hospital, Tongji Medical College, Huazhong University of Science and Technology, Wuhan, China; 7 Biochemistry and Molecular Biology, School of Basic Medicine, Tongji Medical College, Huazhong University of Science and Technology, Wuhan, Hubei China; 8 Cancer Research Institute, Tongji Hospital, Tongji Medical College, Huazhong University of Science and Technology, Wuhan, Hubei 430030 China

**Keywords:** Extracellular matrix, Sequencing, Tumour biomarkers

## Abstract

Discoidin domain receptor 1 (DDR1) is a member of the receptor tyrosine kinase family, and its ligand is collagen. Previous studies demonstrated that DDR1 is highly expressed in many tumors. However, its role in hepatocellular carcinoma (HCC) remains obscure. In this study, we found that DDR1 was upregulated in HCC tissues, and the expression of DDR1 in TNM stage II-IV was higher than that in TNM stage I in HCC tissues, and high DDR1 expression was associated with poor prognosis. Gene expression analysis showed that DDR1 target genes were functionally involved in HCC metastasis. DDR1 positively regulated the migration and invasion of HCC cells and promoted lung metastasis. Human Phospho-Kinase Array showed that DDR1 activated ERK/MAPK signaling pathway. Mechanically, DDR1 interacted with ARF6 and activated ARF6 through recruiting PSD4. The kinase activity of DDR1 was required for ARF6 activation and its role in metastasis. High expression of PSD4 was associated with poor prognosis in HCC. In summary, our findings indicate that DDR1 promotes HCC metastasis through collagen induced DDR1 signaling mediated PSD4/ARF6 signaling, suggesting that DDR1 and ARF6 may serve as novel prognostic biomarkers and therapeutic targets for metastatic HCC.

## Introduction

Hepatocellular Carcinoma (HCC) is the fourth leading cause of cancer-related mortality worldwide [1]. Although surgical treatment is still considered as a curable treatment with many advances for HCC, the five-year survival rate of patients with HCC is only 18% [1, 2]. The long-term prognosis of patients with hepatocellular carcinoma is dismal, due to the high recurrence rate and metastasis rate [3]. Therefore, a detailed study on the molecular mechanism of recurrence and metastasis of hepatocellular carcinoma is necessary.

Discoidin domain receptor 1 (DDR1) is a member of receptor tyrosine kinase family with natural collagen as its specific ligand. DDR1 could therefore interact with extracellular matrix (ECM) through binding with collagen [4]. Although DDR1 is ubiquitously expressed in epithelial cells, the expression level of DDR1 is significantly increased in tumor tissues such as colorectal cancer, breast cancer, lung cancer, glioma, ovarian cancer and esophageal cancer [5–12]. Previous studies showed that DDR1 is involved in key cellular processes, including cell proliferation, migration, survival, and differentiation [13]. Existing models suggest that the kinase activity of DDR1 plays a predominant role in tumorigenesis. For example, Nilotinib inhibited DDR1 kinase activity and reduced the invasion and metastasis of colorectal cancer cells [14]. DDR1 inhibition induced GBM cell autophagy for therapy sensitization [15]. In pancreatic ductal adenocarcinoma, pharmacological inhibition of DDR1 with a small molecule 7rh slowed the tumor progression and enhanced the therapeutic response to standard-of-care PDA regimens [14]. In response to genotoxic stress, tyrosine phosphorylated DDR1 inhibits apoptosis in cells with wild-type p53 in colorectal cancer [15]. However, other studies suggest that the kinase activity is not necessary for the function of DDR1 in the cancer development. DDR1 promoted collective cancer-cell migration through DDR1–Par3/Par6 complex independent of its kinase activity or collagen binding [16]. The invasion of breast cancer cells is regulated by DDR1, and DDR1 kinase activity is not required for invadosome formation or activity [17]. DDR1 regulates multi-organ site metastatic reactivation of breast cancer by non-canonical signaling independent of its kinase activity [18]. However, the role and detailed mechanism of DDR1 in the development of HCC, especially metastatic HCC, remain obscure and incomplete.

Here, we illustrated that DDR1 bound with ADP ribosylation factor 6 (ARF6), a member of the ARF family and the Ras superfamily of small GTP-binding proteins. Previous studies reported that ARF6 localizes in the plasma membrane and endosomes [19, 20] and recycles between GTP-bound (active) and GDP-bound (inactive) forms that are regulated by guanine nucleotide exchange factors (GEFs) and GTPase-activating proteins (GAPs) [19]. There are 10 common human GEFs baring the Sec7 domain known as ArfGEF domain, including CYTH1, CYTH2, CYTH3, CYTH4, GEP100, IQSEC3, PSD, PSD2, PSD3, PSD4 [21, 22]. ARF6 plays important roles in biological processes such as actin cytoskeletal rearrangements and membrane trafficking [19, 20]. It has been reported that ARF6 is involved in cancer cell proliferation, angiogenesis, invasion, and metastasis [21–25]. However, the role of ARF6 in HCC remain unclear.

In this study, we demonstrated the functional impact of DDR1 in promoting migration, invasion and lung metastasis in HCC through collagen induced DDR1 signaling mediated PSD4/ARF6 signaling axis. This molecular mechanism could serve as a novel therapeutic target for clinical application in patients with metastatic HCC, which merits further investigation.

## Results

### DDR1 plays a prometastatic role in HCC in vitro and in vivo

**Fig. 1:**
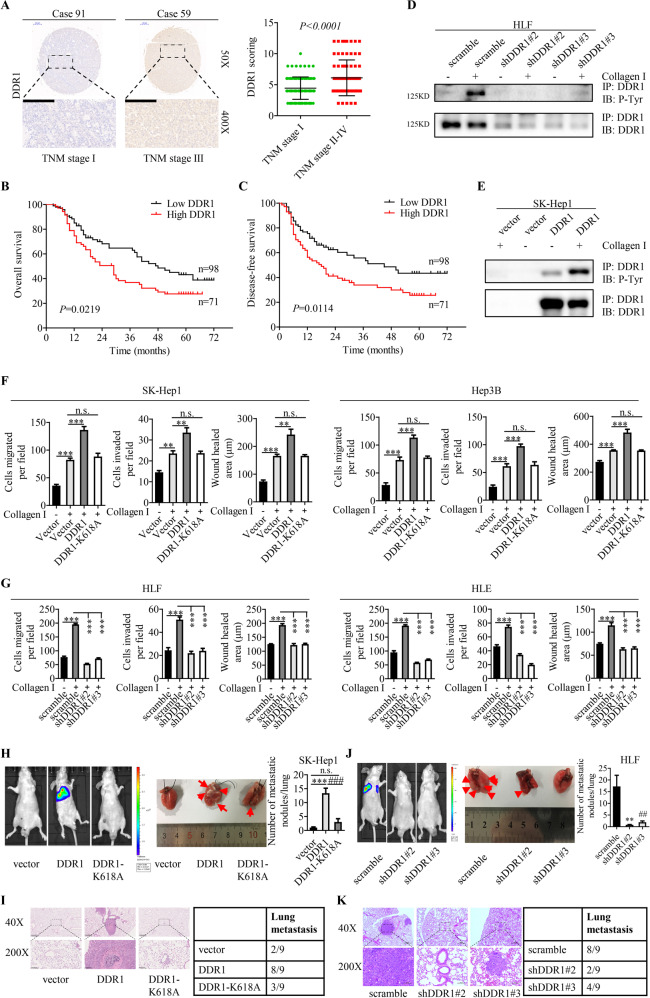
DDR1 plays a prometastatic role in HCC in vitro and in vivo. **A** Immunohistochemical staining (IHC) and expression scoring of DDR1 was performed in 169 HCC tissues. Representative pictures were shown (scale bar: 200 μm). **B**, **C** Kaplan–Meier analysis was used to illustrate the correlation between DDR1 expression and overall survival or disease-free survival of HCC patients. The cutoff for determining low or high DDR1 expression was the median value. **D**, **E** Indicated cells were treated with (+) or without (−) collagen I for 3 h, and immunoprecipitation with anti-DDR1. The blots were probed with the indicated antibodies. **F**, **G** Trans-well migration and invasion assays, wound healing assays were performed in indicated cells. **H**, **J** Lung metastasis from nude mice injected with SK-Hep1 and HLF cells by tail veins from both groups killed at 8 weeks, was measured by bioluminescent imaging (BLI), representative images of lung tissue sections, and number of lung metastatic foci in both groups (*n* = 9). **I**, **K** Representative hematoxylin and eosin staining of lung tissue sections, and incidence of lung metastasis in both groups of BALB/c (nu/nu) mice (*n* = 9). *P < 0.05, **P < 0.01, ***P < 0.001. ^#^*P* < 0.05^, ##^*P* < 0.01, ^###^*P* < 0^.^001: the scramble group compared with the shDDR1#3 group.

To study the clinical significance of DDR1 expression in HCC, Online Oncomine dataset was used to analyze the expression of DDR1 in HCC patients. As shown in Supplementary Fig. S1A–C, DDR1 mRNA level was significantly upregulated in HCC tissues compared with normal liver tissues. We then examined the levels of DDR1 in a tissue microarray consisting a group of 169 HCC tissue samples from Tongji hospital with corresponding clinicopathological features (Supplementary Table S1). IHC staining and scoring showed that the levels of DDR1 were elevated in TNM stage II-IV hepatocellular carcinoma tissues in comparison with TNM stage I tissues (Fig. 1A). Through analyzing the clinicopathological features of patients with HCC, we found that high expression of DDR1 was significantly correlated with poor tumor differentiation (*P* = 0.019), incomplete tumor encapsulation (*P* = 0.029), advanced tumor TNM stage (*P* = 0.012), and tumor recurrence (*P* = 0.001) (Supplementary Table S2). Moreover, the increased DDR1 expression was associated with a poor survival rate and higher recurrence rate in our patient cohort (Fig. 1B, C). Multivariate COX regression analysis demonstrated that high DDR1 expression was an independent and significant factor for recurrence and poor survival (Supplementary Table S3). To investigate the role of DDR1 in HCC cells, we examined expression of DDR1 in a panel of human hepatic and HCC cell lines. As shown in Supplementary Fig. S1D, DDR1 expression varied in human hepatic and HCC cell lines. We knocked down DDR1 in HLF and HLE cells that express high levels of DDR1 (shDDR1-2 and shDDR1-3), and overexpressed DDR1 in SK-Hep1, Hep3B, and HCC-LM3 cells with low DDR1 expression. DDR1 expression level was assessed by Q-PCR and Western blot (Supplementary Fig. S1E–J). Upon collagen stimulation, the phosphorylation level of DDR1 was increased in DDR1-overexpressing cells and reduced in DDR1 knocking down cells (Fig. 1D, E and Supplementary Fig. S1K). However, overexpression of DDR1 kinase-dead (DDR1-K618A) mutants [16] showed the lack of DDR1 phosphorylation upon collagen treatment (Supplementary Fig. S1M). These results were consistent with previous findings [8] and demonstrated that collagen stimulation led to DDR1 phosphorylation in HCC cells. We then performed RNA sequencing to investigate genes regulated by DDR1 (*P* < 0.05) (Supplementary Table S4). Pathway analysis showed that the differentially expressed genes upon DDR1 knockdown (Supplementary Fig. S2A) were mainly involved in extracellular matrix structural constituent, proteinaceous extracellular matrix, extracellular matrix, extracellular matrix component, glycosaminoglycan binding and extracellular matrix organization, implying that DDR1 might be involved in tumor invasion and metastasis. To examine the effect of DDR1 in HCC migration and invasion in vitro, we performed wound healing assay, trans-well migration and invasion assay. Interestingly, treatment with collagen I, significantly enhanced the migration and invasion of SK-Hep1, Hep3B, LM3, HLE, HLF HCC cells (Fig. 1F, G and Supplementary Fig. S2D–F). Overexpression of DDR1 further promoted the migration and invasion of SK-Hep1, Hep3B and LM3 HCC cells (Fig. 1F and Supplementary Fig. S2B, C, F), while overexpression of DDR1-K618A mutants could not achieve the effect. Similarly, the knockdown of DDR1 significantly inhibited the migration and invasion of HLE and HLF cells induced by collagen I (Fig. 1G and Supplementary Fig. S2D, E). To further understand whether DDR1 regulates HCC metastasis in vivo, tail vein injection experiment was performed with HCC cell lines. In vivo metastasis assays showed that overexpression of DDR1 promoted lung metastasis of SK-Hep1 cells. Nevertheless, overexpression of DDR1-K618A mutants failed to showing the same result (Fig. 1H, I, Supplementary Fig. S2G). Consistently, knockdown of DDR1 significantly suppressed lung metastasis of HLF cells (Fig. 1J, K). Together, these results demonstrated that DDR1 plays a prometastatic role in HCC in vitro and in vivo, dependently of its kinase activity.

### DDR1 physically interacts and colocalizes with ARF6

**Fig. 2:**
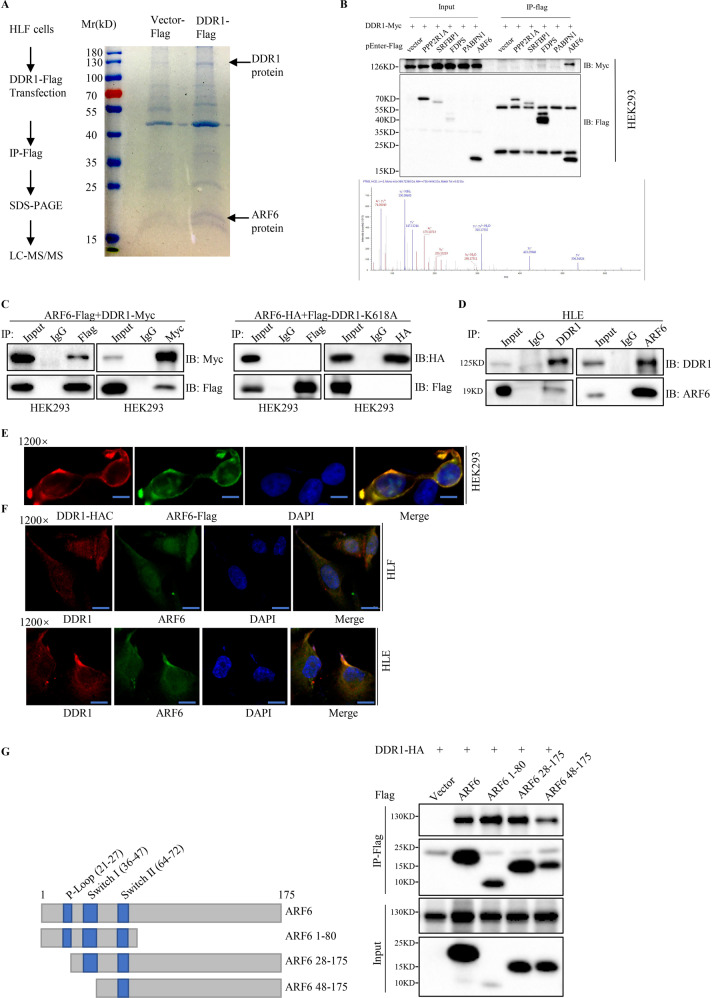
DDR1 physically interacts and colocalized with ARF6. **A** Cellular extracts from SK-Hep1 and HLF cells transfected with FLAG-tagged DDR1 or FLAG-tagged vector were subsequently immunoprecipitated with anti-FLAG antibody. Eluted proteins were separated on SDS-PAGE and visualized by Coomassie blue staining. Eluted proteins were identified by mass spectrometry analysis. **B** Selective genes from mass spectrometry analysis were co-immunoprecipitated with Myc-tagged DDR1 (up). Detection of ARF6 by mass spectrometry (bottom). **C** 293 T cells were transiently co-transfected with indicated plasmids and co-immunoprecipitation assays were performed. **D** Immunoblots of co-immunoprecipitated (IP) endogenous DDR1 and endogenous ARF6 in HLE cell extracts. Immunoglobulin G (IgG) is negative control. **E** Confocal assays were shown to observe the co-localization of exogenously expressed DDR1 and ARF6 in 293 T cells (Scale bar: 15 μm). **F** Confocal assays were shown to observe the co-localization of endogenous DDR1 and ARF6 in HLF and HLE cells (Scale bar: 30 μm). **G** Schematic diagram of FLAG-tagged full-length or deletion constructs of ARF6 used in this study (left panel). Co-precipitation of HA-tagged DDR1 with FLAG-tagged ARF6 or its mutants, analyzed by anti-FLAG immunoprecipitation and anti-FLAG/ HA immunoblots (right panel).

To seek the interacting partners of DDR1 in HCC, we performed mass spectrometry analysis. SK-Hep1 and HLF cells transfected with FLAG-DDR1 or FLAG-vector were immunoprecipitated with anti-Flag antibodies (Fig. 2A). Polypeptides of PPP2R1A, SRFBP1, FDPS, PABPN1 and ARF6 were identified, suggesting these proteins are potential interacting partners of DDR1 (Supplementary Table S5 and 6). In vitro binding assay was used to validate the interaction between DDR1 and its potential interacting candidates. The result showed that DDR1 has strong interaction with ARF6 (Fig. 2B). The interaction was further confirmed by exogenous Co-IP assay in 293 T cells transiently transfected with ARF6-Flag and DDR1-Myc (Fig. 2C). To examine whether the interaction depends on DDR1 kinase activity, we introduced DDR1-K618A for Co-IP assay. The results suggested that the interaction between ARF6 and DDR1 was dependent of DDR1 kinase activity (Fig. 2C). Endogenous Co-IP assay in HLE cells further verified their interaction (Fig. 2D). Moreover, we also detected co-localization of exogenous DDR1 and ARF6 in 293 T cells by immunofluorescence and laser confocal assay (Fig. 2E). Co-localization of endogenous DDR1 and ARF6 in HLF and HLE cells was found (Fig. 2F). To determine the region in ARF6 that mediated DDR1-ARF6 interaction, we performed exogenous Co-IP assay in 293 T cells using various FLAG-tagged ARF6 deletion mutants. The result suggested that aa 28–47 of AFR6 (a region that contains the guanine nucleotide binding switch I) are required for the interaction with DDR1 (Fig. 2G). Taken together, we identified ARF6 as a DDR1 binding partner and the interaction between ARF6 and DDR1 was required for the switch I domain of ARF6.

### DDR1 promotes ARF6 activation in a kinase activity-dependent manner, and promotes MAPK signaling pathway activation

**Fig. 3:**
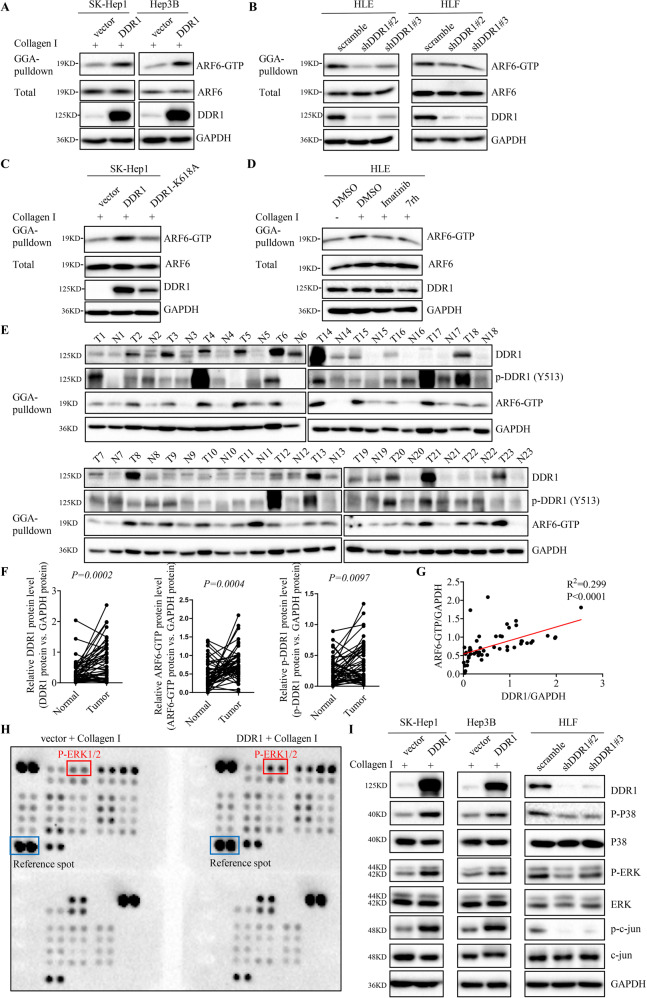
DDR1 promotes ARF6 activation in a kinase activity-independent manner, and promotes MAPK signaling pathway activation. **A** Analysis of the activation level of ARF6 in SK-Hep1 and Hep3B cell lines with collagen I. **B** Analysis of the activation level of ARF6 in HLE and HLF cell lines stably knocked down of DDR1, compared with the scramble groups. **C** Analysis of the activation level of ARF6 in SK-Hep1 cell lines stably overexpressed DDR1, DDR1-K618A, compared with the vector groups with collagen I. **D** Analysis of the activation level of ARF6 in HLE cell lines, treated with or without imatinib, 7rh, and collagen I. **E** The protein or phosphorylation level of DDR1 and ARF6-GTP was analyzed in 50 paired HCC tissues (tumor, T) with corresponding adjacent non-cancerous tissues (normal, N) by western blot. Representative western blot results were shown. **F** Statistical analysis showed DDR1, phosphorylated DDR1 and ARF6-GTP upregulated in HCC tissues, compared with that in normal liver, where GAPDH was used as a control. **G** Spearman correlation analysis between ARF6-GTP and DDR1 expression (*N* = 50). **H** Representative images of human phosphor-kinase array analysis in indicated SK-Hep1 cells with collagen I. The boxed dots are reference protein (blue) and phosphorylated ERK (red). **I** Western blot analysis of the phosphorylation level of P38, ERK and c-jun in indicated HCC cells.

We next asked whether DDR1 could regulate ARF6 expression. Western blot and Q-PCR results showed that overexpression of DDR1 had no effect on ARF6 mRNA or protein levels in SK-Hep1, Hep3B, and LM3 cells in the presence or absence of collagen I treatment (Supplementary Fig. S3A, B, E, F). Similarly, knockdown of DDR1 had no effect on ARF6 mRNA or protein levels in HLF and HLE cells with or without collagen I treatment (Supplementary Fig. S3C, D). Previous studies suggest that activated ARF6 plays important role in tumor progression [21–23, 26, 27], we wondered whether DDR1 could regulate ARF6 activation. By using GGA-pull down assay, we found that overexpression of DDR1 induced the level of ARF6-GTP in SK-Hep1 and Hep3B cells with collagen I treatment (Fig. 3A). Similarly, knocking down of DDR1 reduced ARF6-GTP levels in HLE and HLF cells (Fig. 3B). To further investigated whether DDR1-mediated ARF6 activation depends on DDR1 kinase activity, we used both genetic and pharmacological strategies, including imatinib and 7rh, two DDR1 kinase inhibitors as described in previous studies [18, 28]. Both overexpression of DDR1-K618A and treating the cells with imatinib or 7rh could compromise collagen I-induced ARF6 activation (Fig. 3C, D). Therefore, the activation of ARF6 by DDR1 is dependent of DDR1 kinase activity. To examine the clinical relevance of DDR1 and ARF6 in HCC, we evaluated the protein and phosphorylation levels of DDR1 and ARF6-GTP in a cohort of 50 paired HCC tissues and peripheral non-tumor tissue samples from Tongji hospital by western blot. The results indicated that DDR1, phosphorylated DDR1 and ARF6-GTP were significantly upregulated in tumor tissues compared with corresponding adjacent non-tumor tissues (Fig. 3E, F and Supplementary Fig. S3G). Moreover, our results indicated that DDR1 expression was positively correlated with ARF6-GTP level in HCC tissues (Fig. 3G). Human Phosphor-Kinase Array was applied to examine the relative levels of phosphorylation of 43 kinase phosphorylation protein sites. The results demonstrated that overexpression of DDR1 in SK-Hep1 cells obviously increased the phosphorylation of ERK1/2 with collagen I treatment, compared with other phosphorylation protein sites (Fig. 3H). Moreover, ERK1/2 phosphorylation plays an important role in the metastasis of HCC [29]. We further examined the potential impact of DDR1 on MARK signaling in HCC. The results showed that overexpression of DDR1 enhanced the phosphorylation level of P38, ERK and c-jun in SK-Hep1 and Hep3B cell lines with collagen I stimulation. Consistently, knocking down of DDR1 reduced the phosphorylation level of P38, ERK and c-jun in HLF cell lines (Fig. 3I). Taken together, our results suggested that DDR1 promoted the activation of ARF6 in HCC cells, which was dependent of its kinase activity, and DDR1 activated MAPK signaling.

### Active ARF6 plays a prometastatic role in HCC progress in vitro and in vivo, and promotes MAPK signaling pathway activation

**Fig. 4:**
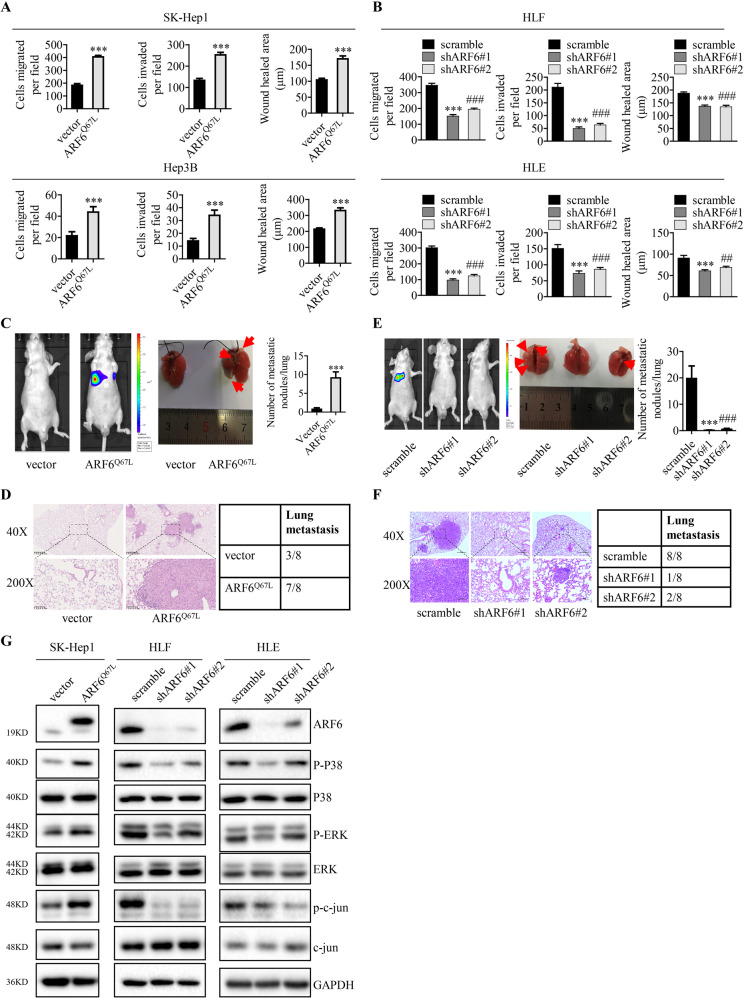
Active ARF6 plays a prometastatic role in HCC progress in vitro and in vivo, and promotes MAPK signaling pathway activation. **A**, **B** Trans-well migration and invasion assays, wound healing assays were performed in indicated cells. **C**, **E** Lung metastasis from nude mice injected with SK-Hep1 and HLF cells by tail veins from both groups killed at eight weeks, was measured by bioluminescent imaging (BLI), representative images of lung tissue sections, and number of lung metastatic foci in both groups (*n* = 8). **D**, **F** Representative hematoxylin and eosin staining of lung tissue sections, and incidence of lung metastasis in both groups of BALB/c (nu/nu) mice (*n* = 8). **G** Western blot analysis of the phosphorylation level of P38, ERK and c-jun in indicated HCC cells. *P < 0.05, **P < 0.01, ***P < 0.001. ^#^*P* < 0.05^, ##^*P* < 0.01, ^###^*P* < 0.001: the scramble group compared with the shARF6#2 group.

To tested whether ARF6 is also involved in HCC development, we used online Oncomine dataset to analyze the expression of ARF6 in HCC patients. As shown in Supplementary Fig. S4A, B, ARF6 mRNA level was significantly upregulated in HCC tissues compared with normal liver tissues. We also analyzed the correlation between ARF6 expression and prognosis of HCC patients in The Cancer Genome Atlas (TCGA) database. Kaplan-Meier analysis revealed that high ARF6 expression was significantly correlated with poor survival of HCC patients (Supplementary Fig. S4C). We then examined the expression levels of ARF6 in a panel of human hepatic and HCC cell lines. As shown in Supplementary Fig. S4D, ARF6 and ARF6-GTP were expressed in various degrees in human hepatic and HCC cell lines. Correlation analysis indicated that DDR1 expression was positively correlated with ARF6-GTP level instead of ARF6 level in human hepatic and HCC cell lines (Supplementary Fig. S4E). We then knocked down ARF6 in HLF and HLE cells which express high level of ARF6, and we stably overexpressed ARF6^Q67L^ (constitutively active ARF6) in SK-Hep1, Hep3B, and Huh7 cells, which express comparably low levels of ARF6. Q-PCR and Western blot validated ARF6 knockdown efficiency or overexpression levels (Supplementary Fig. S4F–I). To examine the effect of ARF6 on the migration and invasion of hepatocellular carcinoma cells in vitro, we performed wound healing assay, trans-well migration and invasion assay. Overexpression of ARF6^Q67L^ significantly promoted the migration and invasion of SK-Hep1, Hep3B and Huh7 cells in vitro (Fig. 4A and Supplementary Fig. S5A, B, C), while knocking down of ARF6 inhibited the migration and invasion of HLE and HLF cells (Fig. 4B and Supplementary Fig. S5A, B). More importantly, tail vein injection of these HCC cells showed that overexpression of ARF6^Q67L^ greatly enhanced lung metastasis of SK-Hep1 HCC cells (Fig. 4C, D and Supplementary Fig. S4J) and knocking down of ARF6 suppressed lung colonization of HLF cells (Fig. 4E, F, and Supplementary Fig. S4J), suggesting that the activated ARF6 promotes HCC metastasis. To determine whether ARF6-regulated HCC progression is associated with MAPK signaling activation, we analyzed MAPK activity. Overexpression of ARF6^Q67L^ enhanced phosphorylation of P38, ERK and c-jun in SK-Hep1 cells. In contrast, knocking down of ARF6 reduced phosphorylation of P38, ERK and c-jun, without changing total P38, ERK and c-jun levels in HLF and HLE cells (Fig. 4G). Overall, these results demonstrated that ARF6 contributed to the migration, invasion and metastasis of hepatocellular carcinoma cells, which is associated to the activation of MAPK signaling.

### DDR1 promotes the migration, invasion and metastasis of HCC cells through ARF6

**Fig. 5:**
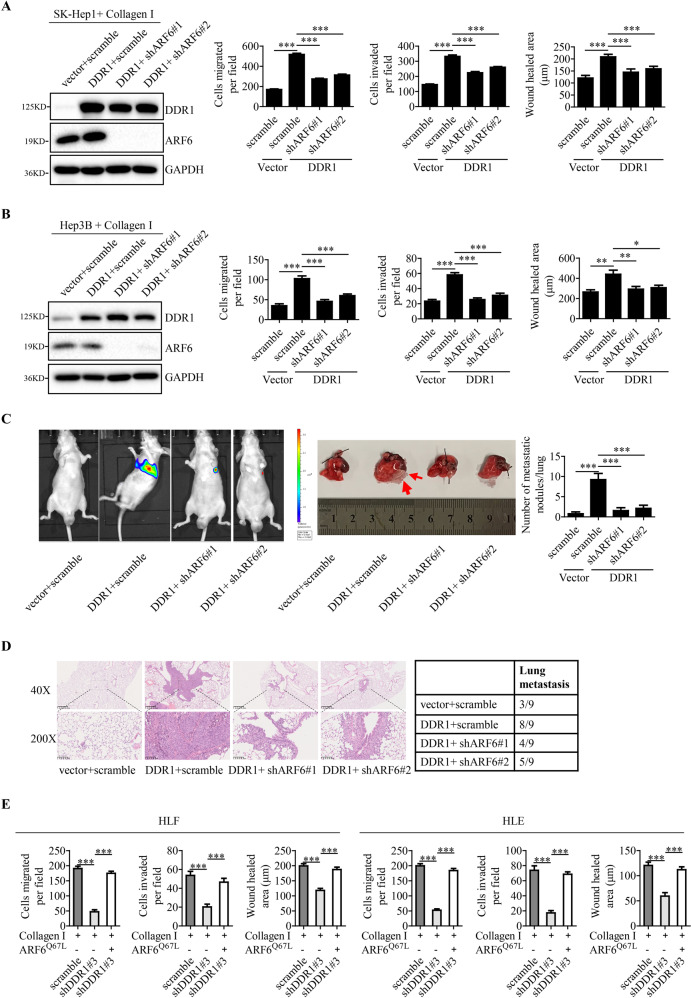
DDR1 promotes the migration, invasion and metastasis of HCC cells through ARF6. DDR1-overexpressing SK-Hep1 (**A**) and Hep3B (**B**) cells were stably transfected with scramble or shARF6 (shARF6#1 and shARF6#2) plasmids to generate control (vector+scramble), DDR1-overexpressing (DDR1 + scramble), and DDR1-overexpressing but ARF6-knockdown (DDR1 + shARF6#1 and DDR1 + shARF6#2) stable cells. **A**, **B**, **E** Trans-well migration and invasion assays, wound healing assays were performed in indicated cells. **C** Lung metastasis from nude mice injected with SK-Hep1 and HLF cells by tail veins from both groups killed at 8 weeks, was measured by bioluminescent imaging (BLI), representative images of lung tissue sections, and number of lung metastatic foci in both groups (*n* = 9). **D** Representative hematoxylin and eosin staining of lung tissue sections, and incidence of lung metastasis in both groups of BALB/c (nu/nu) mice (*n* = 9).

To ask whether the function of DDR1 in HCC depends on ARF6, we knocked down ARF6 in DDR1-overexpressing SK-Hep1 and Hep3B cells. DDR1 and ARF6 expression levels were assessed by Western blot analysis (Fig. 5A, B). Depletion of ARF6 partially abolished tumor cells migration, invasion and metastasis driven by DDR1 overexpression with collagen I (Fig. 5A–D and Supplementary Fig. S5D, E). Similarly, in DDR1-knocked down HLF and HLE cells, overexpressed ARF6^Q67L^ greatly rescued the inhibitory effects of tumor cells migration and invasion when DDR1 silenced (Fig. 5E and Supplementary Fig. S5F, G). The results suggested that ARF6 is one of the essential pathways required for DDR1-mediated HCC progression.

### DDR1 recruits PSD4 for activating ARF6

**Fig. 6:**
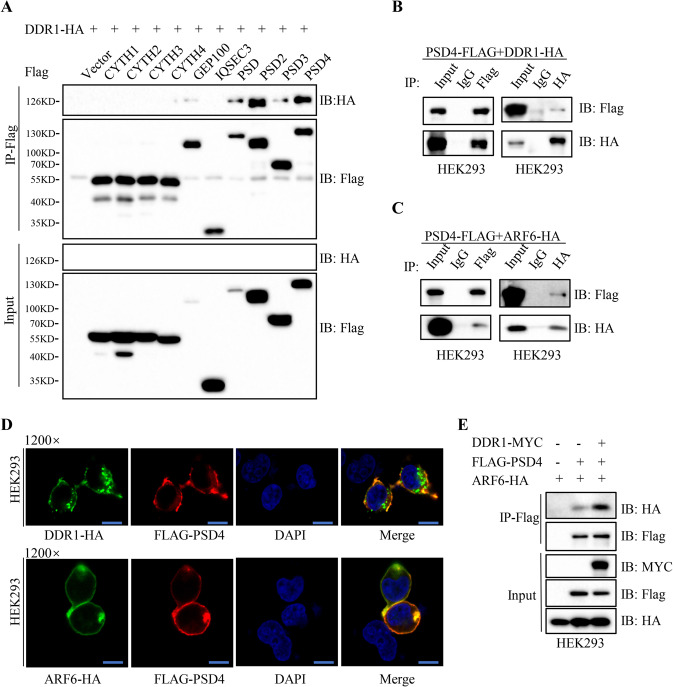
DDR1 recruits PSD4 to activating ARF6. **A** 293 T cells were transiently co-transfected with FLAG-tagged ARF-GEFs and HA-tagged DDR1. Co-immunoprecipitation of DDR1 by ARF-GEFs was shown. **B** 293 T cells were transiently co-transfected with FLAG-tagged PSD4 and HA-tagged DDR1 and co-immunoprecipitation assays were performed. **C** 293 T cells were transiently co-transfected with FLAG-tagged PSD4 and HA-tagged ARF6 and co-immunoprecipitation assays were performed. **D** Confocal assays were shown to observe the co-localization of exogenously expressed PSD4 and DDR1 or ARF6 in 293 T cells (Scale bar 15 μm). **E** 293 T cells were transiently co-transfected with FLAG-tagged PSD4 and HA-tagged ARF6 with or without MYC-tagged DDR1. Co-immunoprecipitation of ARF6 by PSD4 was shown.

As a small GTP-binding protein, ARF6 recycles between GTP-bound and GDP-bound forms that are regulated by guanine nucleotide exchange factors (GEFs) and GTPase-activating proteins. We have showed that DDR1 could induce ARF6-GTP levels in HCC cells (Fig. 3A, B). As ARF-GEF is required for the activation of ARF6, we hypothesize that DDR1 activates ARF6 through recruiting the GEF proteins. Thus, we strived to identify the ARF-GEF that is responsible for DDR1-mediated the activation of ARF6. Ten common human proteins baring the Sec7 domain known as ArfGEF domain were tested [19, 30]. We performed Co-IP in 293 T cells to confirm the interactions between DDR1 and the 10 candidate GEF proteins. The result showed that DDR1 had strong interaction with PSD2 and PSD4 (Fig. 6A). It has been suggested that PSD4 is ubiquitously expressed while the expression of PSD2 is restricted to neuronal cells [31], we then focused on the role of PSD4 in HCC. The DDR1-PSD4 interaction and ARF6-PSD4 interaction were further validated by exogenous Co-IP assay in 293T cells (Fig. 6B, C). Moreover, we showed that PSD4 co-localized with DDR1 or ARF6 in 293 T cells (Fig. 6D), further suggesting that DDR1, ARF6 and PSD4 could form a complex which might contribute to the activation of ARF6. Because DDR1 could bind with PSD4 or ARF6, respectively, we speculated that DDR1 may serve as an adaptor protein to facilitate the interaction of PSD4 with ARF6. HEK293 cells were co-transfected with or without Myc-DDR1, together with Flag-PSD4 and HA-ARF6. We found that overexpression of DDR1 enhanced the interaction between PSD4 and ARF6 (Fig. 6E). To sum up, our results demonstrated that DDR1 recruited PSD4 to ARF6, which could be responsible for the activation of ARF6.

### High PSD4 level predicts poor prognosis in HCC patients, and PSD4 promotes HCC metastasis

**Fig. 7:**
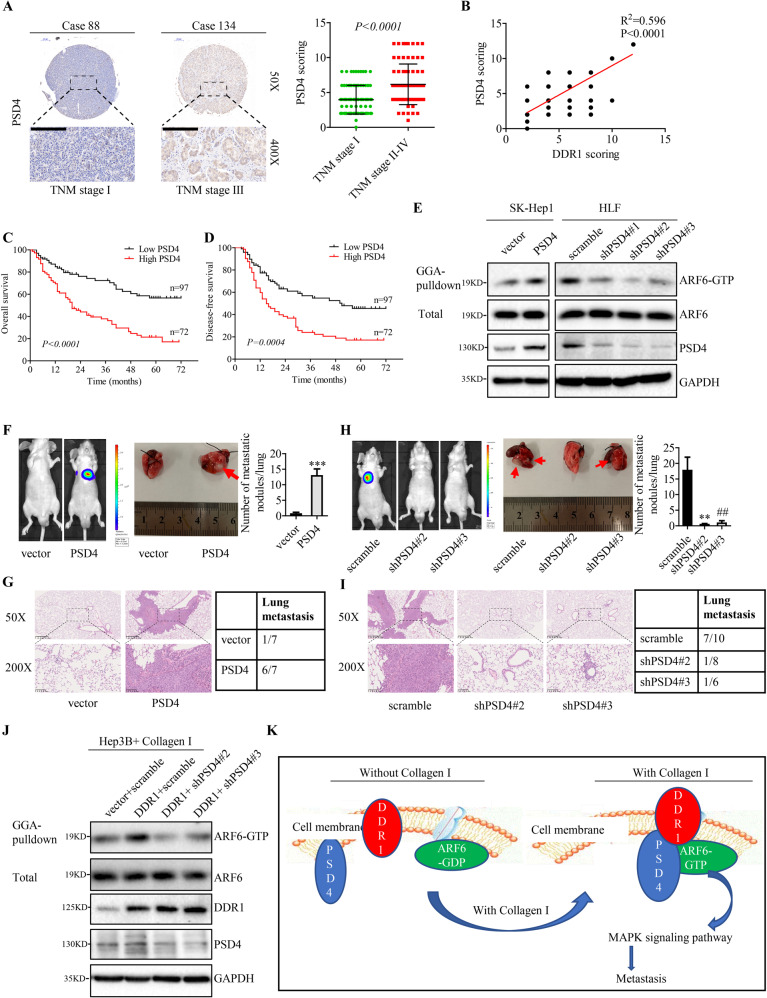
PSD4 plays a prometastatic role in HCC progress in vitro and in vivo. **A** Immunohistochemical staining (IHC) and expression scoring of PSD4 was performed in 169 HCC tissues. Representative pictures were shown (scale bar: 200 μm). **B** Spearman correlation analysis between PSD4 and DDR1 expression. *N* = 169 (right panel). **C**, **D** Kaplan–Meier analysis was used to illustrate the correlation between PSD4 expression and overall survival or disease-free survival of HCC patients. The cutoff for determining low or high PSD4 expression was the median value. **E** Analysis of the activation level of ARF6 in indicated SK-Hep1 and HLF cells. **F**, **H** Lung metastasis from nude mice injected with SK-Hep1 and HLF cells by tail veins from both groups killed at 8 weeks, was measured by bioluminescent imaging (BLI), representative images of lung tissue sections, and number of lung metastatic foci in both groups. **G**, **I** Representative hematoxylin and eosin staining of lung tissue sections, and incidence of lung metastasis in both groups of BALB/c (nu/nu) mice. **J** Analysis of the activation level of ARF6 in indicated Hep3B cells with collagen I. **K** Proposed oncogenic DDR1/PSD4/ARF6 signaling pathway in HCC metastasis. *P < 0.05, **P < 0.01, ***P < 0.001; ^#^*P* < 0.05^, ##^*P* < 0.01, ^###^*P* < 0.001: the scramble group compared with the shPSD4#3 group.

To study the clinical significance of PSD4 in HCC, we investigated its expression levels in a tissue microarray involving 169 HCC samples and then performed a survival analysis with corresponding clinicopathological features. Immunohistochemical staining showed that PSD4 expressed in higher levels in TNM stage II-IV HCC tissues than in TNM stage I tissues (Fig. 7A). Moreover, our results indicated that PSD4 expression was positively correlated with DDR1 expression in HCC samples (Fig. 7B). Combined with clinicopathological analysis of patients with HCC, high expression of PSD4 was associated with poor tumor differentiation (*P* = 0.031), incomplete tumor encapsulation (*P* = 0.001), advanced tumor TNM stage (*P* = 0.001), and tumor recurrence (*P* = 0.001) (Supplementary Table S7). In addition, Kaplan–Meier analysis demonstrated that patients with high PSD4 expression correlated with lower overall survival rate and higher recurrence chance (Fig. 7C, D). These results suggested a role of PSD4 in promoting HCC development. GGA-pull down assay showed that knocking down of PSD4 led to decreased ARF6-GTP level in HLF cells, and overexpression of PSD4 induced GTP bound ARF6 in SK-Hep1 cells (Fig. 7E). To study the function of PSD4 in HCC, we first analyzed its expression levels in a panel of human hepatic and HCC cell lines. As shown in Supplementary Fig. S6A, PSD4 was expressed to varying degrees in human hepatic and HCC cell lines. we knocked down PSD4 in HLF and Huh7 cells that express high levels of PSD4, and overexpressed PSD4 in SK-Hep1 and Hep3B cells expressing comparable low levels of PSD4. PSD4 expression were assessed by Q-PCR and Western blot analysis (Supplementary Fig. S6B–E). Analyzing the MAPK signaling activity in HCC cells also suggested that PSD4 could promote the phosphorylation level of P38, ERK and c-jun (Supplementary Fig. 6F, G). Overexpression of PSD4 enhanced the migration and invasion of Hep3B, SK-Hep1 cells in vitro (Supplementary Fig. S7A, B, E, F), while depletion of PSD4 inhibited the migration and invasion of Huh7 and HLF cells (Supplementary Fig. S7C, D, G, H). To investigate whether PSD4 is involved in HCC metastasis, we injected SK-Hep1 cells into nude mice via tail vein injection and found that overexpression of PSD4 greatly enhanced lung metastasis (Fig. 7F, G, and Supplementary Fig. S7I). In contrast, knocking down of PSD4 suppressed lung metastasis of HLF cells (Fig. 7H, I and Supplementary Fig. S7I). These results together suggested that PSD4 promoted the migration, invasion, and metastasis of HCC cells. To further understand whether PSD4 is required for DDR1-mediated ARF6 activation, we performed GGA-pull down assay with collagen I treatment. The depletion of PSD4 comprised ARF6 activation induced by overexpressed DDR1 (Fig. 7J). Collectively, our results suggested that PSD4 regulates DDR1-mediated activation of ARF6, which is essential for HCC metastatic progression. Altogether, our results illustrated that DDR1 promoted the migration, invasion and metastasis of HCC cells through collagen induced DDR1 signaling mediated recruitment of PSD4 to ARF6, thereby leading to a sustained ARF6 and MAPK signaling activation status (Fig. 7K).

## Discussion

Metastasis is a leading cause of high recurrence and mortality in hepatocellular carcinoma (HCC). However, the underlying mechanism remains obscure. Previous findings indicate that DDR1 is upregulated in colorectal cancer, breast cancer, lung cancer, glioma, ovarian cancer and esophageal cancer, and is involved in tumor invasion and metastasis [5–10]. Some studies demonstrate that DDR1 promotes tumor progression, such as tumor cells migration, invasion, and metastasis [32, 33]. Genetic and pharmacological inhibitions for targeting DDR1 obtains an effective therapy [34–36]. In our study, we found that DDR1 was upregulated in HCC tissues, and the expression of DDR1 in TNM stage II-IV was higher than that in TNM stage I in HCC tissues. High DDR1 expression was also associated with poor prognosis. Multivariate analysis indicated that DDR1 expression was an independent and significant risk factor for recurrence and survival. Detailed Cell function and mice model studies demonstrated that DDR1 played a crucial role in the metastasis of hepatocellular carcinoma. RNA sequencing and pathway analysis indicated that DDR1 regulated genes involved in tumor invasion and metastasis. Both in vitro and in vivo assays suggested that DDR1 positively regulated the migration and invasion of HCC cells and promoted lung metastasis. Also, we found that DDR1 could activated MAPK signaling. Together, our data demonstrate that DDR1 exerts a tumor-promoting role in HCC progression, and could be a potential therapeutic target for HCC metastasis.

To study how DDR1 functions in the invasion and metastasis of HCC, we applied mass spectrometry analysis and identified ARF6 as a novel DDR1 binding partner. DDR1 induces ARF6-GTP levels dependent of its kinase activity in HCC cells, which might be responsible for DDR1-mediated HCC metastasis. Our results suggested the function of DDR1 in HCC metastasis which is both collagen and its kinase activity dependent. Further study showed that DDR1 activated ARF6 through recruiting PSD4, which is collagen dependent. Our results reveal that the DDR1/PSD4/ARF6 signaling axis acts as an important inducer in the regulation of the migration, invasion and lung metastasis of hepatocellular carcinoma cells by collagen induced DDR1 signaling. Besides PSD4, we also found that DDR1 can interact with several other ARF-GEFs. Our data could not exclude the possibility that other GEF proteins are also involved in DDR1-mediated ARF6 activation. Moreover, our results could not exclude a kinase activity-independent role for DDR1 in HCC metastasis, which might function in parallel to the DDR1/PSD4/ARF6 signaling. Indeed, previous studies have suggested a kinase-independent function of DDR1 in tumors [16–18]. Further investigation will be needed to study the function of DDR1 as a non-canonical receptor signaling in HCC metastasis.

ADP ribosylation factor 6 (ARF6) is a member of the ARF family, which belongs to the Ras superfamily of small GTP-binding proteins. ARF6 could be activated by ARF-guanine nucleotide exchange factors (ARF-GEFs). Previous studies demonstrated that ARF6 plays important roles in tumor proliferation, angiogenesis, invasion, and metastasis [21–23, 26, 27]. However, no evidence suggests whether ARF6 is involved in hepatocellular carcinoma. In our study, we showed that ARF6 promotes the migration, invasion and lung metastasis of hepatocellular carcinoma cells. Moreover, ARF6 is required for DDR1-mediated tumor cell migration and invasion, suggesting that ARF6 and DDR1 function together to regulate the development of HCC. Previous studies suggested that ARF6 could act as a mediator of the recycling and endocytosis of multiple membrane receptors, including GNAQ and GPCRs [37–40]. It will be interesting to know whether ARF6 could regulate the recycling and endocytosis of DDR1.

Pleckstrin and Sec7 domain containing 4 (PSD4) is a member of the EFA6 family. EFA6 consists of four isoforms (EFA6A, EFA6B, EFA6C, and EFA6D), which are encoded by PSD, PSD4, PSD2, and PSD3, respectively. EFA6A, B, and D express ubiquitously whereas the EFA6C expression is restricted in neuronal cells. It has been reported that EFA6B inhibits tight junction disassembly and loss of epithelial polarity, and functions as a tumor suppressor at the early stages of breast cancer [31]. While other findings indicate that EFA6B promotes the invasion and metastasis of renal cancer through ARF6 activation mediated by Lysophosphatidic acid [41]. These studies indicate that the role of EFA6B in tumors might be context dependent. In this study, we have demonstrated that EFA6B promotes the migration and invasion of hepatocellular carcinoma cells, and is required for DDR1-mediated ARF6 activation. Collectively, our results suggested that PSD4 functions in accordance with DDR1 and ARF6 for regulating HCC development, which is a novel functional and molecular mechanism and a possible therapeutic target for HCC metastasis.

Altogether, our results illustrated DDR1, ARF6 and PSD4 promoted the migration, invasion and metastasis of HCC cells in vitro and in vivo, we also discovered that DDR1 promoted HCC metastasis through DDR1 signaling mediated PSD4/ARF6 signaling. Concretely, DDR1 interacted with ARF6 and PSD4 to facilitate the recruitment of PSD4 to ARF6 by collagen induced DDR1 signaling, thereby leading to a sustained ARF6 and MAPK signaling activation status, and enhancing migration, invasion and metastasis of HCC cells (Fig. 7K).

## Materials and methods

### Tissue specimens and Immunohistochemistry

From January 2006 to December 2012, 169 samples of human HCC tissues were obtained from patients who underwent surgical tumor resection at Hepatic Surgery Center, Tongji Hospital, Huazhong University of Science and Technology, P.R. China. All HCC tissues were identified in Tongji hospital’s department of pathology. Before surgery, there was no local or systemic therapy. After surgery, no anticancer treatment was conducted before recurrence. Written informed consent was obtained from all patients and each procedure was approved by the Ethical Committee of Tongji Hospital. Tumor staging was made in accordance with the Sixth Edition of Tumor-Node-Metastasis (TNM) Classification of International Union Against Cancer [42]. HCC samples (*n* = 169) were used for producing a tissue microarray (Shanghai Biochip Co., Ltd. Shanghai, China). The fundamental processes of the Immunohistochemistry assay have been mentioned previously [24]. The image scores were evaluated by three different pathologists ignorant of patient clinical pathological characteristics. The total score of each image was calculated by multiplying the score of staining area percentage by intensity score as mentioned previously [24]. Concretely, the percentage of positive cells in each spot from the tissue microarray was divided into five levels (percentage scores): 0 (<10%); 1 (10–25%); 2 (26–50%); 3 (51–75%); and 4 (>75%). The staining intensity was divided into four levels (intensity scores): 0 (negative); 1 (light brown); 2 (brown); and 3 (dark brown) (Supplementary Fig. S1D). The cutoff for determining low or high expression was the median value.

### Reagents and antibodies

Rat tail Collagen I (BD Bioscience, MA, USA), DDR1 inhibitor imatinib and 7rh (MedChemExpress, NJ, USA). Puromycin, trypsin-EDTA, Opti-MEM medium and polybrene were obtained as previous described [25]. G418 was obtained from promoter company (promoter, Wuhan, China). Lipofectamine 2000 Reagent and Lipofectamine 3000 Reagent were purchased from Invitrogen (Life Technologies, Carlsbad, CA, USA). All antibodies used in the project were detailed in Supplementary Table S8.

### Cell lines and Culture

Human fetal liver cell line HL-7702, HCC cell lines Alex, HLF, SK-Hep1, HLE, Hep3B, Huh7, MHCC-97H, MHCC-LM3 and Bel7402 were obtained from the Hepatic Surgery Center, Tongji Hospital, Huazhong University of Science and Technology, P.R. China. 293 T cells were purchased from the China Center for Type Culture Collection (Wuhan, China). The cell lines were cultivated in Dulbecco’s Modified Eagle’s Medium (DMEM, Gibco, ThermoFisher Scientific, Waltham, Massachusetts, USA) supplemented with 10% fetal bovine serum (FBS, Gibco, North America) at 37 °C in 5% CO_2_ and 95% air.

### Plasmids

The human DDR1 (NM_001954.4) cDNA, and pcDNA3.1 plasmid were gifts from the Hepatic Surgery Center, Tongji Hospital, Huazhong University of Science and Technology, P.R. China. pBABE-puro (Plasmid #1764), gag/pol (Plasmid #14887), pMD2.G(Plasmid #12259), pLKO.1 - TRC cloning vector (Plasmid # 10878), psPAX2(Plasmid #12260) were purchased from Addgene (Addgene, Cambridge, MA, USA). To establish pBABE-Flag-DDR1 or pBABE-Flag-ARF6^Q67L^ or pBABE-Flag-PSD4 plasmid, the human cDNA was cloned into the BamHI/EcoRI site of the pBABE-puro retroviral vector, and was identified by sequencing (TSINGKE, Wuhan, China). To construct pLKO.1-scramble, pLKO.1-shDDR1, pLKO.1-shARF6, and pLKO.1-shPSD4 plasmid, the target double-stranded oligonucleotides (shRNA) sequences and one non-targeting sequence (negative control, scramble) were annealed and cloned into the AgeI/EcoRI site of pLKO.1 vector. The sequences of target shRNA oligo pairs are listed in Supplementary Table S9. Viral production, infection, establishment of stable cell clones were described previously [43]. pcDNA3.1 plasmid inserted by Flag-, Myc- or HA- tagged DDR1 and its mutants, Flag- or HA-tagged ARF6 and PSD4, Flag-tagged PPP2R1A, SRFBP1, FDPS, PABPN, CYTH1, CYTH2, CYTH3, CYTH4, GEP100, IQSEC3, PSD, PSD2, PSD3 were constructed according to ClonExpress II One Step Cloning Kit and Mut Express II Fast Mutagenesis Kit V2(Vazyme, Nanjing, China) protocol and were identified by sequencing (TSINGKE, Wuhan, China).

### Coomassie blue staining and mass spectrometry

293 T cells transiently transfected with FLAG-DDR1 or FLAG-vector were lysed in IP lysis buffer (25 mM Tris-HCl (pH 7.4), 150 mM NaCl, 1% NP-40, 1 mM EDTA, 10% Glycerol and protease inhibitor cocktails) and IP assays were performed as described previously [43]. The eluted proteins were separated by SDS-PAGE followed by Coomassie blue staining. There was a significant difference in the gel bands between the FLAG-DDR1 group and FLAG-vector group among the molecular weight 15kd-25kd region. Mass spectrometry was detected and analyzed by ptm-bio lab (PTM BIO, Hangzhou, China).

### Immunofluorescence

Immunofluorescence assay was performed as described previously [43]. In brief, after the indicated treatments, cells were cultured on coverslips for 12 h, fixed in 4% paraformaldehyde for 15 min at room temperature, and permeabilized with 0.5% Triton X-100 for 20 min. After blocking, the slides were incubated with according primary antibody overnight at 4 °C in a humidified box. After that, the slides were then washed three times and incubated with according secondary antibody for 4 h at room temperature in a humidified box. Finally, cell nuclei were stained by 40, 60-diamidino-2-phenylindole (DAPI, Sigma-Aldrich) for 5 min. Pictures were obtained by phase-contrast and confocal laser-scanning microscopy.

### Immunoblotting, co-immunoprecipitation (co-IP)

Immunoblotting assay and co- immunoprecipitation assay were performed as described previously [43]. Briefly, cells were collected and lysed on ice with IP lysis buffer. lysates were incubated with protein G agarose for 2 h, and immunoprecipitated with indicated antibodies at 4 °C overnight. Then lysates were incubated with protein G agarose for 1 h followed by 1wash using IP lysis buffer and 3 washes using washing buffer (300 mM NaCl, 1.0 mM EDTA, 25 mM Tris-HCl, pH7.4, 1.0% NP-40). The beads were eluted with 2×SDS-PAGE loading buffer and then subjected to immunoblotting analysis.

### Reverse transcription PCR and Real-time quantitative PCR

Total cell RNA was extracted with TRIzol Reagent (Invitrogen, Life Technologies, Carlsbad, CA, USA). Reverse transcription was carried out with the QuantScript RT Kit (TIANGEN, Beijing, China) according to manufacturers’ introductions [43]. Real-time fluorescence quantitative PCR was performed with the CFX96 Touch™ Real-Time PCR Detection System (Bio-Rad, Hercules, CA, USA) using SuperReal PreMix Plus (SYBR Green) kit (TIANGEN, Beijing, China) according to the manufacturer’s protocol [43]. Each gene expression level was normalized to that of glyceraldehyde-3-phosphate dehydrogenase (GAPDH) of the same sample. Each sample was done in triplicate independently. The primers are listed in Supplementary Table S10.

### Wound healing assay

Adhered cells were seeded in 6-well plates and cells grew to 95% or 100% confluence. Monolayer cells were scratched using a 10 μL pipette tip to form a wound. Cells migrating into the scratched filed were recorded by phase contrast microscopy (DM400B, Leica Corporation, Germany) at 24 h after the scratch. Photographs of cells migrating into the scratched filed were taken for analysis of migration ability.

### Trans-well migration and invasion assay

We investigated the migration and invasion of HCC cells with a 24-well trans-well plate containing 8-μm pores (Corning, MA, USA). For migration assays, 5 × 10^4^ cells in 100 μL of serum free DMEM were placed into the upper chamber, 650 μL DMEM containing 10% FBS was placed to the lower chambers. For invasion assays, chamber inserts were pretreated with BD Matrigel (BD Biosciences, NJ, USA) coating (2 mg/ml) overnight. Then, 1 × 10^5^ cells in 200 μL of serum free DMEM were placed into the upper chamber. After culturing for 24 h (migration) or 48 h (invasion), cells migrating towards or invaded the lower chamber were fixed in 4% phosphate-buffered neutral formalin for 15 min, stained with crystal violet for 15 min and counted by bright-field microscopy (DM400B, Leica Corporation, Germany). Photographs of cells migrating towards or invaded the lower chamber were taken for analysis of migration and invasion ability.

### In vivo metastasis assay

Animal assays were carried out according to Wuhan Medical Experimental Animal Care Guidelines. Male BALB/c (nu/nu) mice were bred under specific pathogen-free (SPF) conditions and were used until 6 weeks old. In vivo lung metastasis assay was performed as described previously [44]. DDR1 or ARF6^Q67L^-overexpression stable SK-Hep1 cells produced by retrovirus transduction, and DDR1 or ARF6-knockdown stable HLF cells produced by lentivirus transduction were used. Briefly, 1 × 10^6^ SK-Hep1 and HLF cells were injected into the tail veins of the randomized mice. Bioluminescent imaging was used to observe metastatic outgrowth. 8 weeks after injection, mice were sacrificed, and the lungs were removed, visually examined tumor nodules and taken photos, fixed with formalin, and stained with hematoxylin and eosin.

### ARF6 activation assays

ARF6 activation was examined with the active Arf6 Activation Assay Kit (Cell Biolabs, San Diego, CA, USA). ARF6-GTP pull-downs were carried out according to the manufacturer’s instructions. The theory of the assay is that active ARF6-GTP binds specifically with the protein-binding domain (PBD) of GGA3. To examine the ARF6-GTP level, cells after treatment were harvested with ice-cold 1X Assay/Lysis Buffer for 20 min and then cleared by centrifugation for 10 min (14,000 x g at 4 °C). GGA3 PBD Agarose bead was added to the cleared lysates at 4 °C for 1 h. The level of ARF6-GTP and total ARF6 in the lysates were determined by immunoblotting using Anti-Arf6 Antibody.

### Human phospho-kinase array

Proteome Profiler Array Human Phospho-Kinase Array Kit (ARY003B, R&D Systems, Inc. USA & Canada) was applied to examine the relative levels of different protein phosphorylation according to the manufacturer’s instruction.

### Statistical analyses

Data analysis was performed using Prism 5.0 (GraphPad Software, La Jolla, CA, USA) software. The results were presented as the mean ± SEM. The difference between two groups were analyzed by two-tailed Student’s *t*-test, ANOVA test, or a nonparametric test. χ2 test or Fisher’s exact test was used to analyze categorical data. The survival between subgroups was assessed by Kaplan–Meier and log-rank analysis. Three independent experiments were carried out to insure repeatability. We used a COX proportional hazards model to define the independent factors of survival and recurrence, which were based on the variables elected in univariate and multivariate analyses. A value of *P* < 0.05 was regarded as statistically significance.

### RNA-Seq

Cells were lysed with TRIzol reagent (Invitrogen, Life Technologies, Carlsbad, CA, USA). RNA extraction, library construction, high-throughput sequencing and data analysis were conducted in Novogene Technology Co., Ltd. (Beijing, P.R. China).

## Supplementary information

Supplementary Materials for DDR1 promotes hepatocellular carcinoma metastasis through recruiting PSD4 to ARF6
