# Supplementary material for: DDR1 promotes hepatocellular carcinoma metastasis through recruiting PSD4 to ARF6
Source: Oncogene. 2022 Feb 9;41(12):1821–34. doi: 10.1038/s41388-022-02212-1 (PMC8933278; doi:10.1038/s41388-022-02212-1)
Supplement: Supplementary file 1 — Supplementary Materials for DDR1 promotes hepatocellular carcinoma metastasis through recruiting PSD4 to ARF6 [file 41388_2022_2212_MOESM1_ESM.docx]

Supplementary Materials for

**DDR1 promotes hepatocellular carcinoma metastasis through recruiting PSD4 to ARF6**

**Supplementary Figure S1**


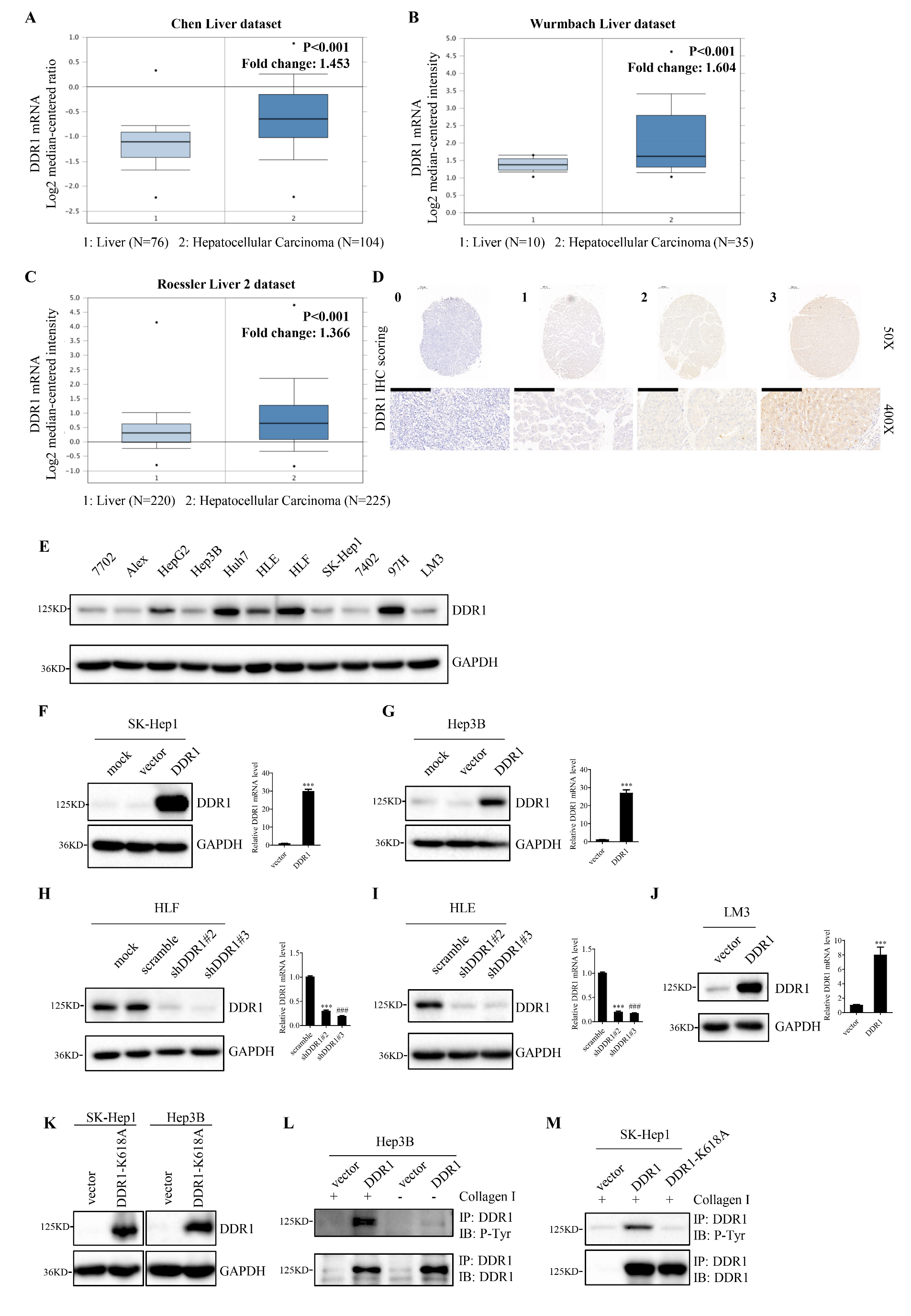


**Supplementary Figure S1. DDR1 protein is significantly upregulated in HCC tissues and DDR1 expression in HCC cells. (A-C)** DDR1 mRNA level in normal liver and hepatocellular carcinoma in the Dataset from Oncomine database (<https://www.oncomine.org>). **(D)** Representative images of DDR1 stained sections with different intensity scores were shown (scale bar: 200μm). **(E)** DDR1 expression level in hepatic and HCC cell lines was analyzed by Western blotting. Western blotting (left panel) and Q-PCR (right panel) analysis of DDR1 expression in SK-Hep1 **(F)**, Hep3B **(G)** and LM3 **(J)** cell lines stably overexpressed vector and DDR1; HLF **(H)** and HLE **(I)** cells lines stably knocked down of scramble and DDR1. **(K)** Western blotting analysis of DDR1 expression in SK-Hep1 (left panel), Hep3B (right panel) cell lines stably overexpressed vector and DDR1-K618A. **(L, M)** Indicated cells were treated with (+) or without (-) collagen Ⅰ for 3 hours, and immunoprecipitation with anti-DDR1. The blots were probed with the indicated antibodies.

**Supplementary Figure S2**


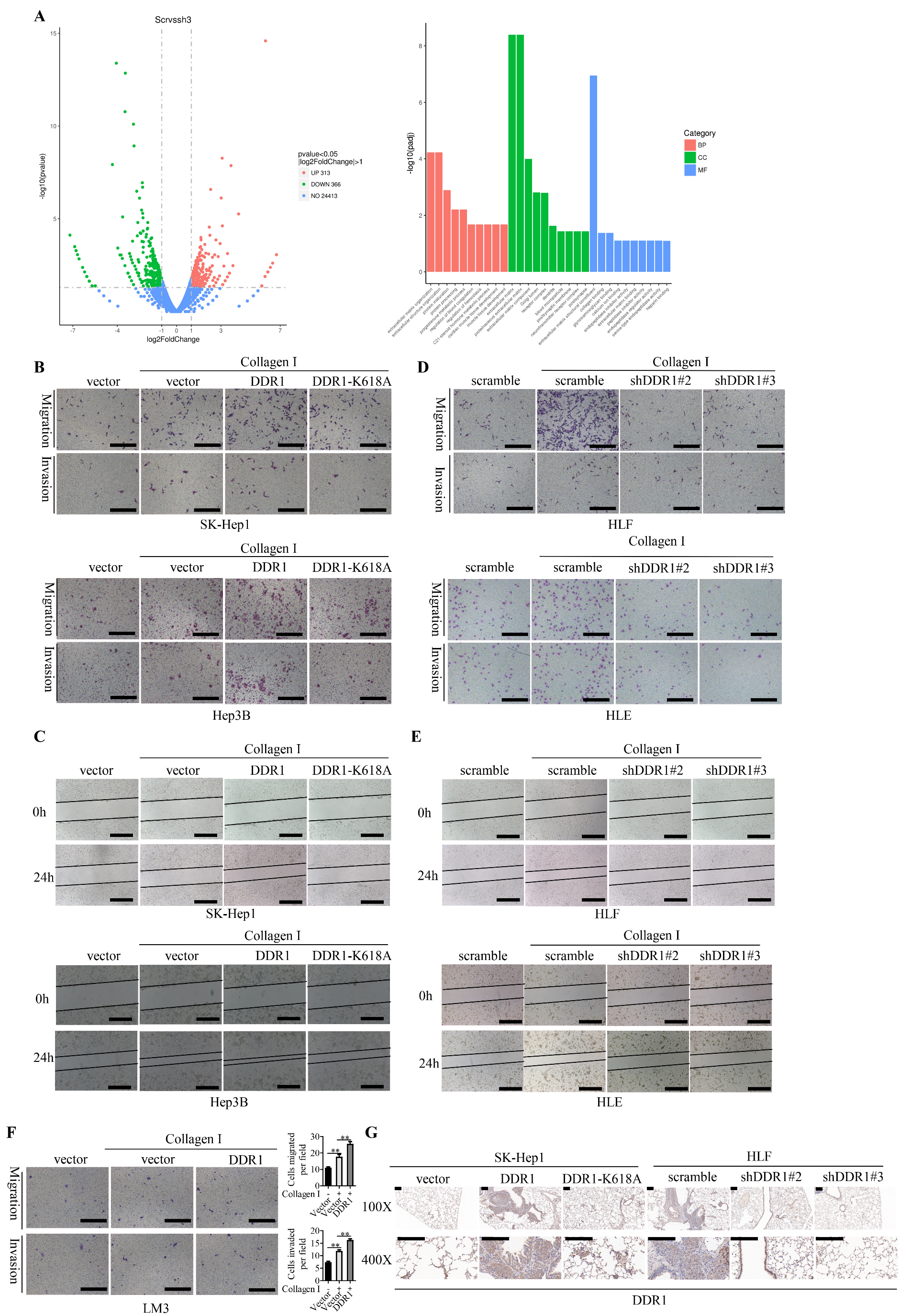


**Supplementary Figure S2. DDR1 plays a prometastatic role in HCC progress in vitro.** **(A)** Top gene ontology terms enriched for genes significantly regulated with shDDR1#3. Padj served as p value after correction of multiple hypothesis test. BP, biological process; CC, cellular component; MF, Molecular Function. **(b, d, F)** Trans-well migration (up) and invasion (bottom) assays in indicates cells (magnification, ×100; scale bar: 50μm). **(C, E)** Wound healing assays in indicates cells (magnification, ×50; scale bar: 500μm). **(G)** IHC for DDR1 in lung tissue from nude mice was performed. Representative pictures were shown (scale bar: 200μm).

**Supplementary Figure S3**


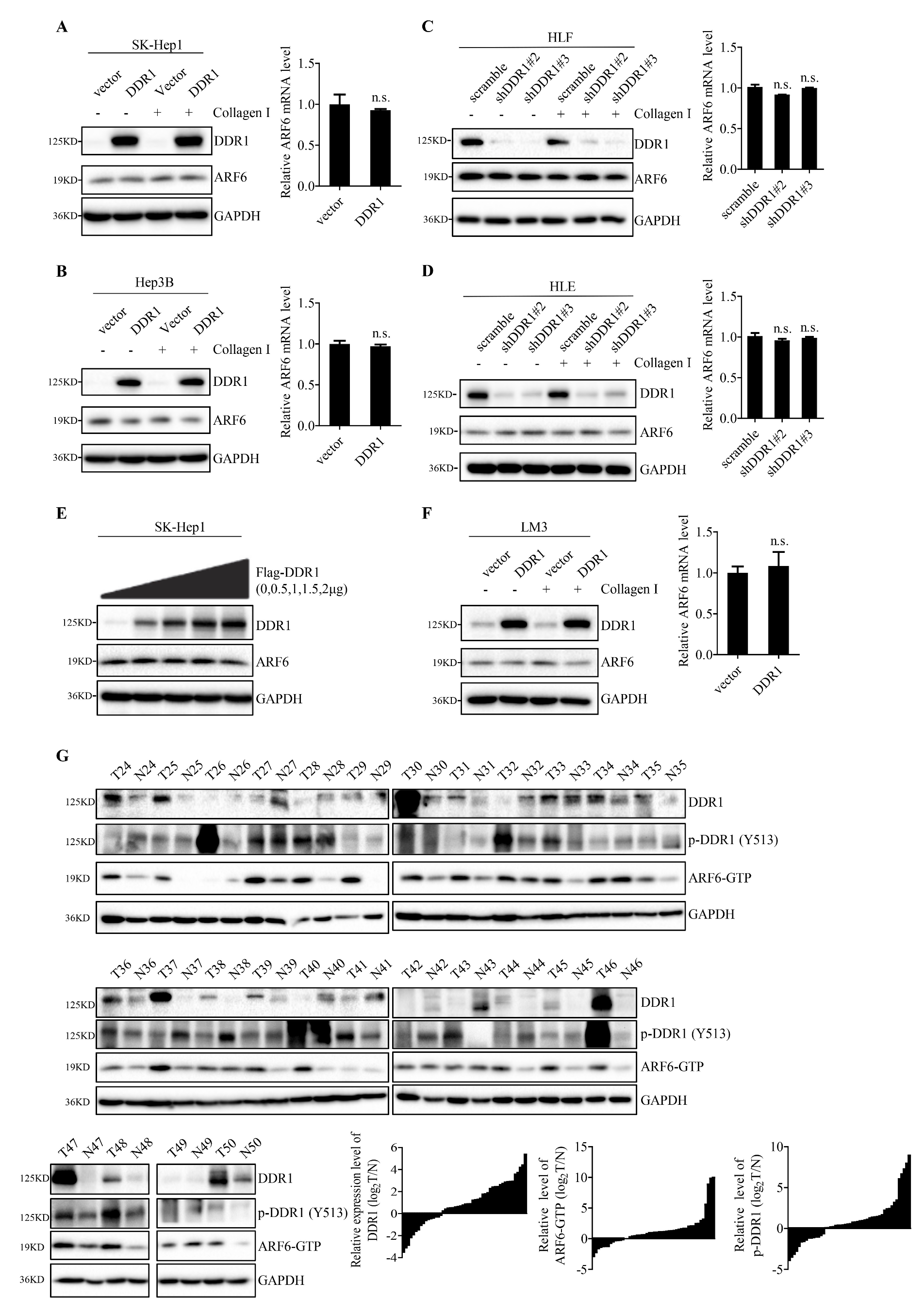


**Supplementary Figure S3. DDR1 has no obvious effect on the expression of ARF6.** Western blotting (left panel) and Q-PCR (right panel) analysis of the level of DDR1 and ARF6 in SK-hep1 **(A)**, Hep3B **(B)** and LM3 **(F)** cell lines stably overexpressed DDR1, compared with the vector groups with or without collagen I; Western blotting (left panel) and Q-PCR (right panel) analysis of the level of DDR1 and ARF6 in HLF **(C)** and HLE **(D)** cells lines stably knocked down of DDR1, compared with the scramble groups with or without collagen I. **(E)** Western blotting analysis of the level of DDR1 and ARF6 in SK-hep1 cell lines transfected with increasing amount of DDR1 plasmids. n.s., no significance. **(G)** Expression level of DDR1, phosphorylated DDR1, and ARF6-GTP in 50 paired HCC tissues (T) with their corresponding non-cancerous tissues (N). Statistical analysis showed that DDR1 and ARF6-GTP bands of HCC tissues were quantified and shown in the bar chart after being normalized to the respective adjacent non-tumor tissues.

**Supplementary Figure S4**

**
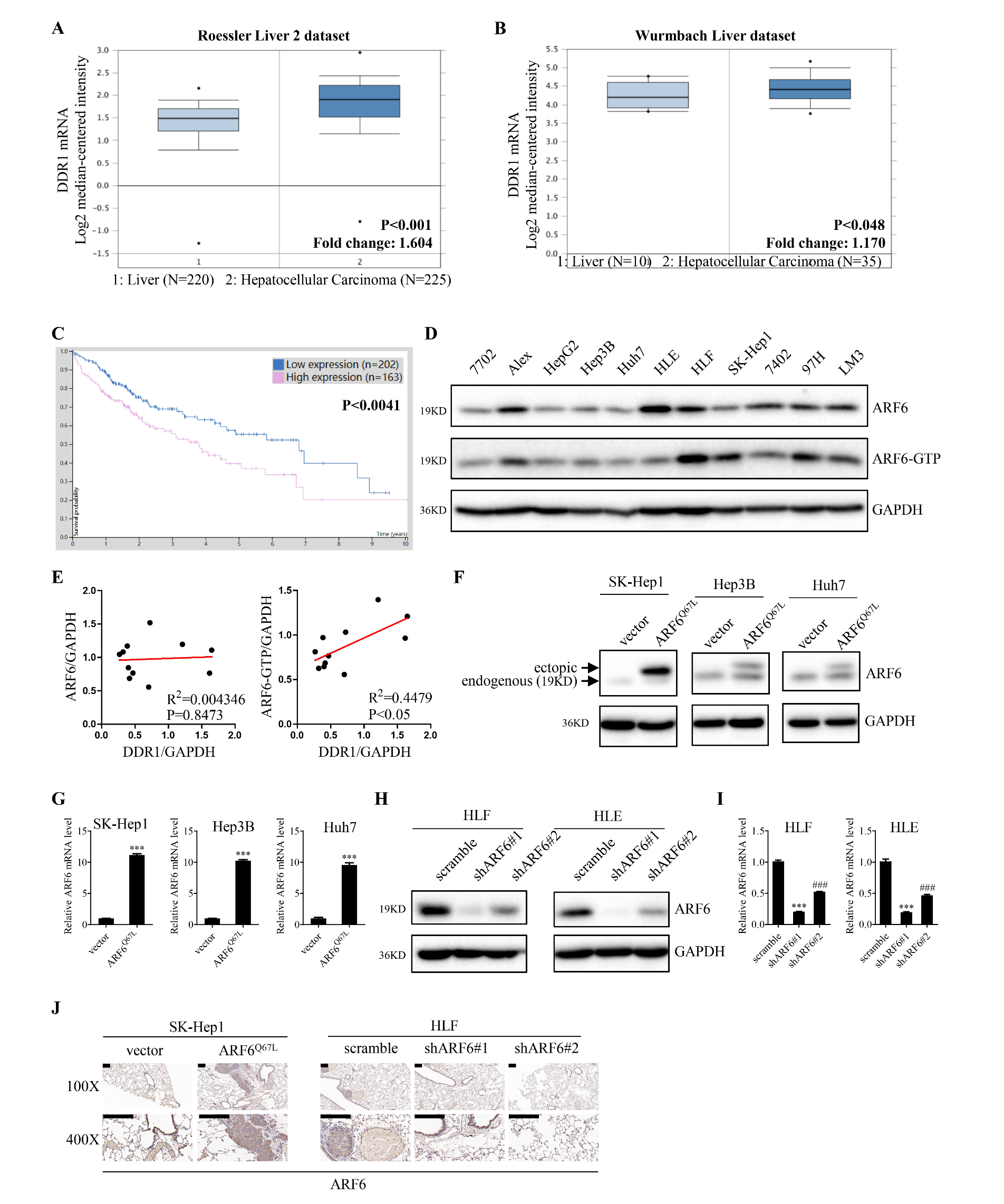
**

**Supplementary Figure S4. ARF6 expression in HCC.** **(A, B)** ARF6 mRNA level in normal liver and hepatocellular carcinoma in the Dataset from Oncomine database (<https://www.oncomine.org>). **(C)** Kaplan-Meier analyses of the correlations between ARF6 mRNA levels and overall survival of 365 patients with HCC. Data were obtained and analyzed at the website (http:// www.proteinatlas.org); the original data could be searched from The Cancer Genome Atlas (TCGA) database. **(D)** ARF6 expression and activation level in hepatic and HCC cell lines was analyzed by Western blotting. **(E)** Spearman correlation analysis between DDR1 expression and ARF6 or ARF6-GTP level in hepatic and HCC cell lines. **(F, G)** Western blotting and Q-PCR analysis of ARF6 expression in SK-Hep1, Hep3B and Huh7 cell lines stably overexpressed vector and ARF6^Q67L^; **(H, I)** HLF and HLE cells lines stably knocked down of scramble and ARF6. **(J)** IHC for ARF6 in lung tissue from nude mice was performed. Representative pictures were shown (scale bar: 200μm).

**Supplementary Figure S5**

**
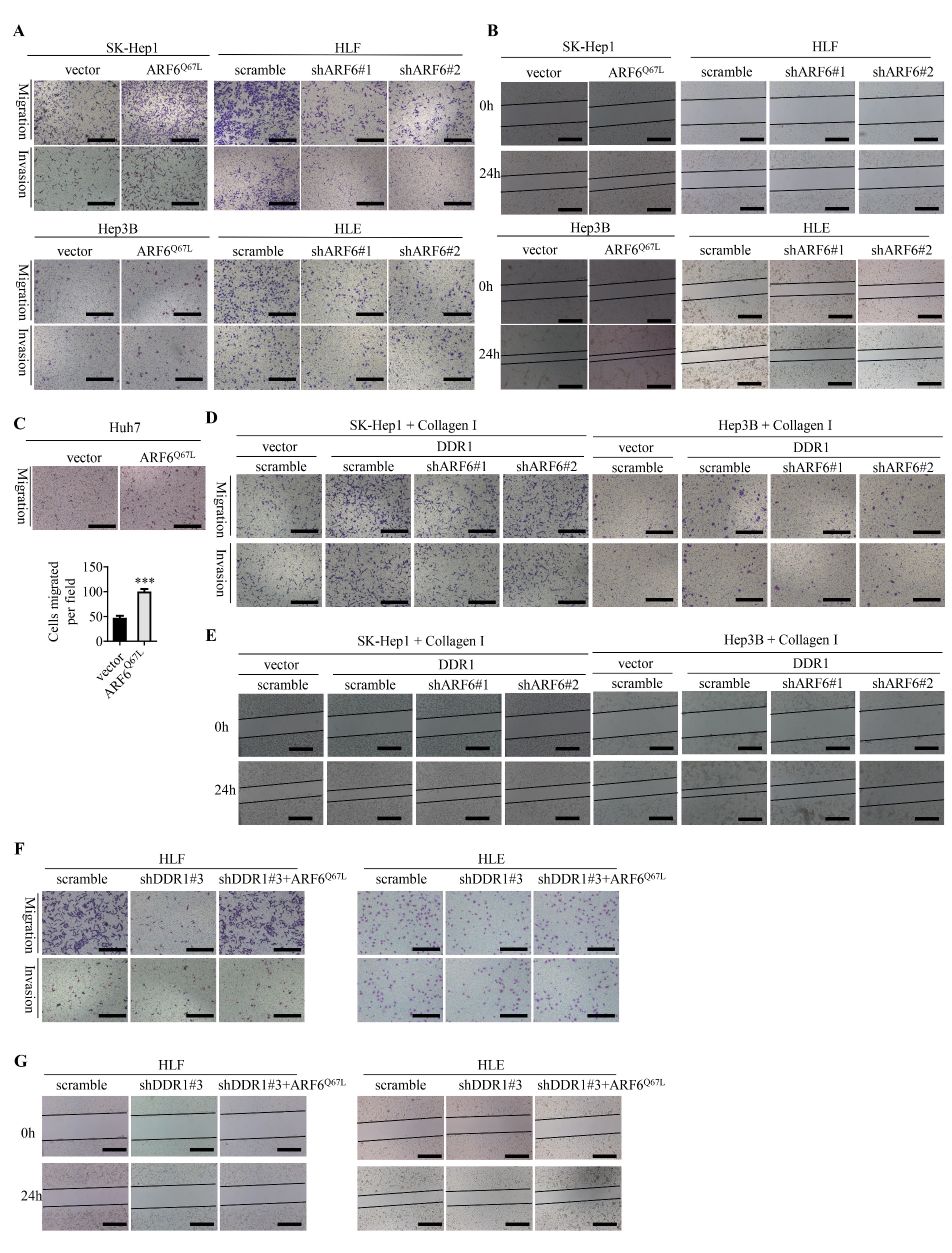
**

**Supplementary Figure S5. Active ARF6 plays a prometastatic role in HCC progress in vitro.** **(a, c, D, F)** Trans-well migration (up) and invasion (bottom) assays in indicates cells (magnification, ×100; scale bar: 50μm). **(B, E, G)** Wound healing assays in indicates cells (magnification, ×50; scale bar: 500μm).

**Supplementary Figure S6**

**
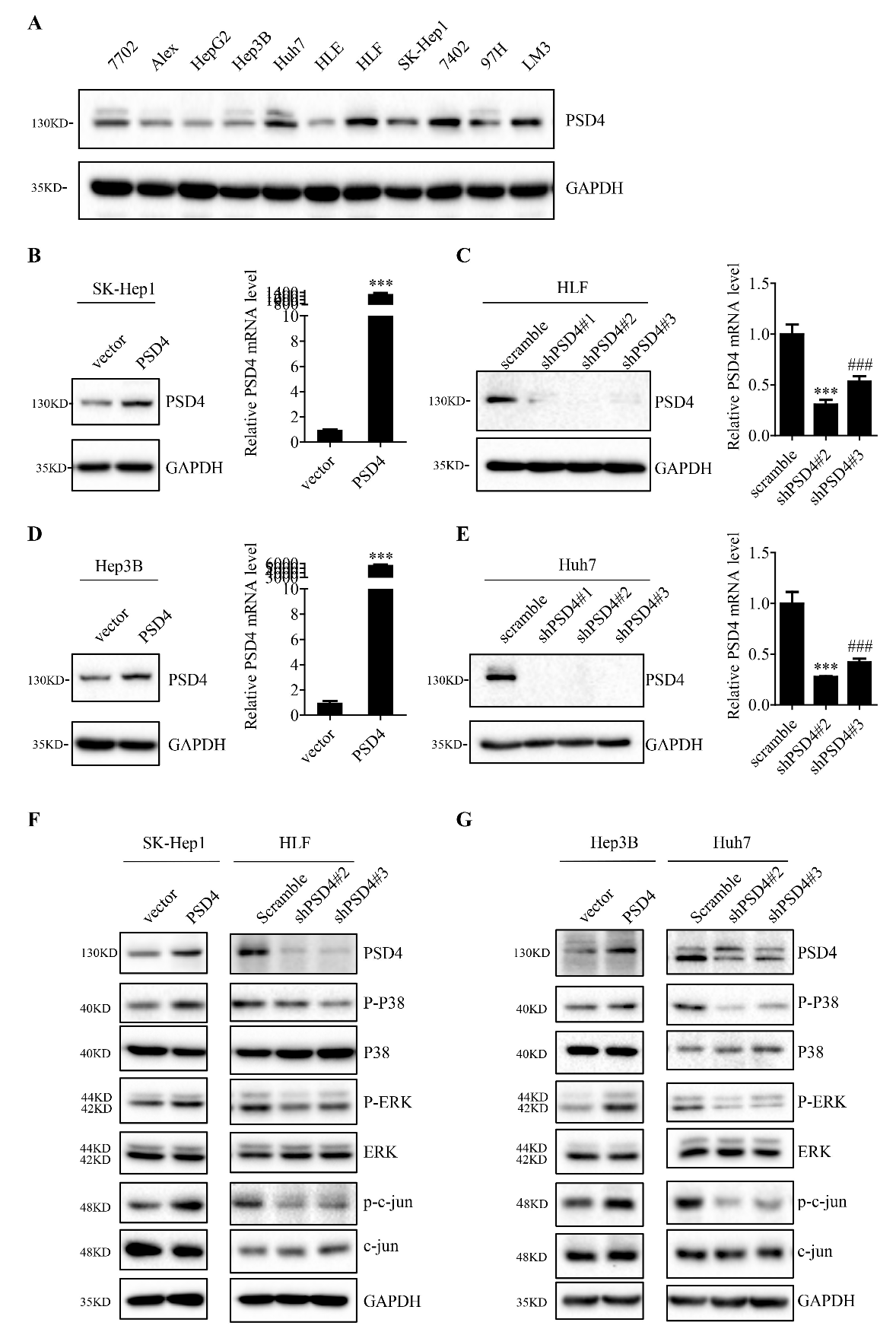
**

**Supplementary Figure S6. PSD4 expression in HCC cells, and its effect on MAPK signaling pathway. (A)** PSD4 expression level in hepatic and HCC cell lines was analyzed by Western blotting. Western blotting (left panel) and Q-PCR (right panel) analysis of PSD4 expression in SK-Hep1 **(B)** and Hep3B **(D)** cells stably overexpressed vector and PSD4; HLF **(C)** and Huh7 **(E)** cells stably knocked down of scramble and PSD4. **(F, G)** Western blotting analysis of the phosphorylation level of P38, ERK and c-jun in indicated HCC cells.

**Supplementary Figure S7**

**
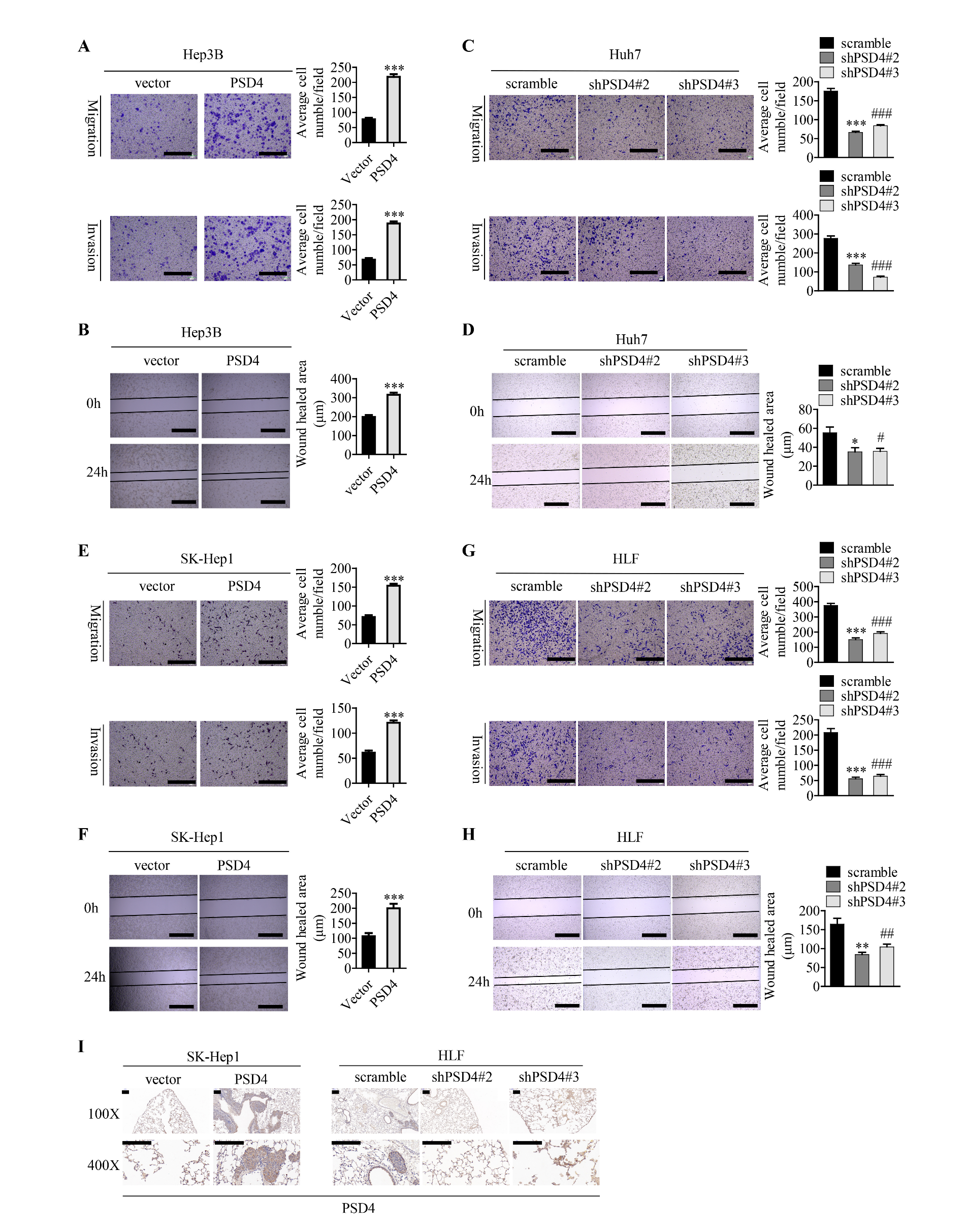
**

**Supplementary Figure S7. PSD4 plays a prometastatic role in HCC progress in vitro.** Trans-well migration (up) and invasion (bottom) assays of Hep3B **(A)**, Huh7 **(C)**, SK-Hep1 **(E)**, and HLF **(G)** cells (magnification, ×100; scale bar: 50μm). Wound healing assays of Hep3B **(B)**, Huh7 **(D)**, SK-Hep1 **(F)**, and HLF **(H)** hepatocellular carcinoma cells (magnification, ×50; scale bar: 500μm). **(I)** IHC for PSD4 in lung tissue from nude mice was performed. Representative pictures were shown (scale bar: 200μm).

| **Supplementary Table S1. Clinicopathologic features of patients with hepatocellular carcinoma.** | | |
| --- | --- | --- |
| **Clinicopathological Features** | **n=169 Number** | **Percentage (%)** |
|  |  |  |
| ***Sex*** |  |  |
| Male | 145 | 85.80 |
| Female | 24 | 14.20 |
| ***Age(years)*** |  |  |
| ≤50 | 87 | 51.48 |
| ＞50 | 82 | 48.52 |
| ***ALT (ng/ml)*** |  |  |
| ≤75 | 137 | 81.07 |
| ＞75 | 32 | 18.93 |
| ***Serum AFP (ng/ml)*** |  |  |
| ＜400 | 92 | 54.44 |
| ≥400 | 77 | 45.56 |
| ***GGT(u/l)*** |  |  |
| ≤54 | 68 | 40.24 |
| ＞54 | 101 | 59.76 |
| ***HBV*** |  |  |
| Negative | 21 | 12.43 |
| Positive | 148 | 87.57 |
| ***HCV*** |  |  |
| Negative | 167 | 98.82 |
| Positive | 2 | 1.18 |
| ***Differentiation*** |  |  |
| Well/moderate | 88 | 52.07 |
| Poor | 81 | 47.93 |
| ***Tumor size(cm)^#^*** |  |  |
| ＜5 | 75 | 44.38 |
| ≥5 | 94 | 55.62 |
| ***Tumor encapsulation*** |  |  |
| Present | 74 | 43.79 |
| Absent | 95 | 56.21 |
| ***Tumor number*** |  |  |
| Single | 133 | 78.70 |
| Multiple | 36 | 21.30 |
| ***TNM stage*** |  |  |
| I | 91 | 53.85 |
| II+III+IV | 78 | 46.15 |
| ***Recurrence*** |  |  |
| Yes | 89 | 52.66 |
| No | 80 | 47.34 |

^#^ Tumor size was measured by the length of the largest tumor nodule.

**Supplementary Table S2. Correlation between DDR1 Expression with Clinicopathologic Features in HCC (n=169).**

| **Clinicopathological Features** | **Total** | **Relative DDR1 Expression** | | ***P* value** |
| --- | --- | --- | --- | --- |
|  |  | **Low** | **High** |  |
| ***Sex*** |  |  |  |  |
| Male | 145 | 85 | 60 | 0.824 |
| Female | 24 | 13 | 11 |  |
| ***Age(years)*** |  |  |  |  |
| ≤50 | 87 | 48 | 39 | 0.533 |
| ＞50 | 82 | 50 | 32 |  |
| ***ALT (ng/ml)*** |  |  |  |  |
| ≤75 | 137 | 81 | 56 | 0.556 |
| ＞75 | 32 | 17 | 15 |  |
| ***Serum AFP (ng/ml)*** |  |  |  |  |
| ＜400 | 92 | 53 | 39 | 1.000 |
| ≥400 | 77 | 45 | 32 |  |
| ***GGT(u/l)*** |  |  |  |  |
| ≤54 | 68 | 40 | 28 | 0.875 |
| ＞54 | 101 | 58 | 43 |  |
| ***HBV*** |  |  |  |  |
| Negative | 21 | 13 | 8 | 0.815 |
| Positive | 148 | 85 | 63 |  |
| ***HCV*** |  |  |  |  |
| Negative | 167 | 97 | 70 | 1.000* |
| Positive | 2 | 1 | 1 |  |
| ***Differentiation*** |  |  |  |  |
| Well/moderate | 88 | 59 | 29 | **0.019** |
| Poor | 81 | 39 | 42 |  |
| ***Tumor size(cm)^#^*** |  |  |  |  |
| ＜5 | 75 | 42 | 33 | 0.754 |
| ≥5 | 94 | 56 | 38 |  |
| ***Tumor encapsulation*** |  |  |  |  |
| Present | 74 | 50 | 24 | **0.029** |
| Absent | 95 | 48 | 47 |  |
| ***Tumor number*** |  |  |  |  |
| Single | 133 | 74 | 59 | 0.259 |
| Multiple | 36 | 24 | 12 |  |
| ***TNM stage*** |  |  |  |  |
| I | 91 | 61 | 30 | **0.012** |
| II+III+IV | 78 | 37 | 41 |  |
| ***Recurrence*** |  |  |  |  |
| Yes | 89 | 40 | 49 | **0.001** |
| No | 80 | 58 | 22 |  |

NOTE. Statistical significance (P <0.05) is shown in bold

^#^ Tumor size was measured by the length of the largest tumor nodule.

*; Fisher exact test.

**Supplementary Table S3. Univariate and multivariate analysis of factors associated with**

**survival and recurrence of 169 HCC patients.**

NOTE. Statistical significance (P <0.05) is shown in bold

| **Supplementary Table S4. Gene microarray results of shDDR1#3 in HLF cells.** | | | | |
| --- | --- | --- | --- | --- |
| Gene_id | log_2_FoldChange | p-value | Gene_name | Regulation |
| ENSG00000169439 | 4.060984 | 4.15E-14 | SDC2 | up |
| ENSG00000168542 | 3.452720 | 1.43E-13 | COL3A1 | up |
| ENSG00000143341 | 3.472080 | 1.69E-11 | HMCN1 | up |
| ENSG00000187134 | 2.904159 | 7.92E-11 | AKR1C1 | up |
| ENSG00000151632 | 2.866532 | 1.17E-09 | AKR1C2 | up |
| ENSG00000137809 | 4.326843 | 1.21E-08 | ITGA11 | up |
| ENSG00000196139 | 2.305382 | 1.16E-07 | AKR1C3 | up |
| ENSG00000197614 | 2.285573 | 2.02E-07 | MFAP5 | up |
| ENSG00000069535 | 2.683436 | 3.31E-07 | MAOB | up |
| ENSG00000071073 | 2.496609 | 7.73E-07 | MGAT4A | up |
| ENSG00000170017 | 2.355620 | 3.40E-06 | ALCAM | up |
| ENSG00000010810 | 2.280291 | 5.09E-06 | FYN | up |
| ENSG00000038427 | 3.635109 | 7.98E-06 | VCAN | up |
| ENSG00000196730 | 2.261445 | 8.91E-06 | DAPK1 | up |
| ENSG00000213694 | 1.936700 | 1.60E-05 | S1PR3 | up |
| ENSG00000153823 | 2.607830 | 1.60E-05 | PID1 | up |
| ENSG00000136869 | 1.945553 | 3.23E-05 | TLR4 | up |
| ENSG00000205670 | 1.971082 | 3.59E-05 | SMIM11A | up |
| ENSG00000141052 | 2.248828 | 4.53E-05 | MYOCD | up |
| ENSG00000172159 | 2.452459 | 5.74E-05 | FRMD3 | up |
| ENSG00000070915 | 2.403959 | 6.15E-05 | SLC12A3 | up |
| ENSG00000078401 | 2.022663 | 6.88E-05 | EDN1 | up |
| ENSG00000139155 | 7.196085 | 7.55E-05 | SLCO1C1 | up |
| ENSG00000268089 | 2.642625 | 8.25E-05 | GABRQ | up |
| ENSG00000163536 | 2.055909 | 9.62E-05 | SERPINI1 | up |
| ENSG00000113594 | 1.672589 | 0.000105 | LIFR | up |
| ENSG00000188243 | 1.727302 | 0.000119 | COMMD6 | up |
| ENSG00000163661 | 2.964862 | 0.000160 | PTX3 | up |
| ENSG00000081052 | 1.867216 | 0.000163 | COL4A4 | up |
| ENSG00000123843 | 2.330704 | 0.000165 | C4BPB | up |
| ENSG00000117228 | 2.233032 | 0.000173 | GBP1 | up |
| ENSG00000213626 | 1.766706 | 0.000202 | LBH | up |
| ENSG00000196586 | 1.761270 | 0.000277 | MYO6 | up |
| ENSG00000143344 | 1.845440 | 0.000291 | RGL1 | up |
| ENSG00000187678 | 6.876615 | 0.000315 | SPRY4 | up |
| ENSG00000232537 | 6.876615 | 0.000315 | AC092810.3 | up |
| ENSG00000157445 | 2.243423 | 0.000339 | CACNA2D3 | up |
| ENSG00000167984 | 3.962159 | 0.000377 | NLRC3 | up |
| ENSG00000165072 | 1.609757 | 0.000422 | MAMDC2 | up |
| ENSG00000249641 | 6.784323 | 0.000517 | HOXC13-AS | up |
| ENSG00000188738 | 6.784323 | 0.000517 | FSIP2 | up |
| ENSG00000272148 | 6.784323 | 0.000517 | AC013403.2 | up |
| ENSG00000272505 | 6.784323 | 0.000517 | AC104964.3 | up |
| ENSG00000259416 | 6.784323 | 0.000517 | AC021739.3 | up |
| ENSG00000179104 | 1.587428 | 0.000560 | TMTC2 | up |
| ENSG00000140092 | 1.694025 | 0.000560 | FBLN5 | up |
| ENSG00000144857 | 2.253085 | 0.000649 | BOC | up |
| ENSG00000271503 | 3.167547 | 0.000740 | CCL5 | up |
| ENSG00000237437 | 3.167547 | 0.000740 | ASS1P12 | up |
| ENSG00000137393 | 1.576372 | 0.000752 | RNF144B | up |
| ENSG00000070190 | 6.685722 | 0.000858 | DAPP1 | up |
| ENSG00000248027 | 3.741328 | 0.000885 | AP001351.1 | up |
| ENSG00000102230 | 3.818755 | 0.000885 | PCYT1B | up |
| ENSG00000103226 | 1.645398 | 0.000947 | NOMO3 | up |
| ENSG00000135643 | 1.479818 | 0.000984 | KCNMB4 | up |
| ENSG00000172000 | 1.533340 | 0.001032 | ZNF556 | up |
| ENSG00000133067 | 1.773377 | 0.001044 | LGR6 | up |
| ENSG00000257285 | 3.103806 | 0.001085 | AL132780.1 | up |
| ENSG00000127129 | 2.002016 | 0.001170 | EDN2 | up |
| ENSG00000172602 | 3.659508 | 0.001370 | RND1 | up |
| ENSG00000123870 | 3.659508 | 0.001370 | ZNF137P | up |
| ENSG00000174028 | 1.612472 | 0.001390 | FAM3C2 | up |
| ENSG00000073464 | 1.586156 | 0.001423 | CLCN4 | up |
| ENSG00000076258 | 6.579885 | 0.001437 | FMO4 | up |
| ENSG00000227374 | 6.579885 | 0.001437 | AL157832.1 | up |
| ENSG00000163295 | 1.427139 | 0.001491 | ALPI | up |
| ENSG00000065320 | 2.587868 | 0.001592 | NTN1 | up |
| ENSG00000207523 | 2.587868 | 0.001592 | SNORA66 | up |
| ENSG00000148677 | 3.037118 | 0.001598 | ANKRD1 | up |
| ENSG00000121068 | 3.037118 | 0.001598 | TBX2 | up |
| ENSG00000144369 | 3.037118 | 0.001598 | FAM171B | up |
| ENSG00000163347 | 1.570788 | 0.001749 | CLDN1 | up |
| ENSG00000113196 | 1.416239 | 0.001784 | HAND1 | up |
| ENSG00000091129 | 1.466688 | 0.001877 | NRCAM | up |
| ENSG00000163017 | 1.576965 | 0.002126 | ACTG2 | up |
| ENSG00000116141 | 2.539185 | 0.002142 | MARK1 | up |
| ENSG00000118785 | 2.539185 | 0.002142 | SPP1 | up |
| ENSG00000197008 | 1.467192 | 0.002147 | ZNF138 | up |
| ENSG00000078018 | 1.374118 | 0.002298 | MAP2 | up |
| ENSG00000198346 | 2.967197 | 0.002365 | ZNF813 | up |
| ENSG00000021300 | 1.859078 | 0.002384 | PLEKHB1 | up |
| ENSG00000172403 | 1.900976 | 0.002412 | SYNPO2 | up |
| ENSG00000067141 | 1.330483 | 0.002435 | NEO1 | up |
| ENSG00000133131 | 1.707845 | 0.002596 | MORC4 | up |
| ENSG00000127324 | 1.579726 | 0.002726 | TSPAN8 | up |
| ENSG00000163328 | 1.501044 | 0.002748 | GPR155 | up |
| ENSG00000197580 | 1.781542 | 0.002863 | BCO2 | up |
| ENSG00000172572 | 1.244194 | 0.003041 | PDE3A | up |
| ENSG00000163362 | 1.428588 | 0.003221 | INAVA | up |
| ENSG00000120738 | 1.707433 | 0.003301 | EGR1 | up |
| ENSG00000255546 | 3.480477 | 0.003346 | AP001973.1 | up |
| ENSG00000177675 | 2.816287 | 0.003515 | CD163L1 | up |
| ENSG00000106034 | 1.799129 | 0.003553 | CPED1 | up |
| ENSG00000261428 | 2.059491 | 0.003603 | AC097461.1 | up |
| ENSG00000131711 | 1.213521 | 0.003667 | MAP1B | up |
| ENSG00000186868 | 1.308690 | 0.003737 | MAPT | up |
| ENSG00000273301 | 1.561277 | 0.003814 | AC016717.2 | up |
| ENSG00000183691 | 1.482245 | 0.003840 | NOG | up |
| ENSG00000238078 | 6.341616 | 0.004174 | LINC01352 | up |
| ENSG00000141338 | 6.341616 | 0.004174 | ABCA8 | up |
| ENSG00000256195 | 6.341616 | 0.004174 | AP002518.1 | up |
| ENSG00000140795 | 1.213609 | 0.004265 | MYLK3 | up |
| ENSG00000279806 | 1.440232 | 0.004332 | AC018629.1 | up |
| ENSG00000125864 | 1.509340 | 0.004650 | BFSP1 | up |
| ENSG00000105767 | 1.311543 | 0.004697 | CADM4 | up |
| ENSG00000119681 | 1.212568 | 0.005178 | LTBP2 | up |
| ENSG00000175600 | 2.734467 | 0.005247 | SUGCT | up |
| ENSG00000258469 | 2.734467 | 0.005247 | CHMP4BP1 | up |
| ENSG00000123838 | 2.108839 | 0.005291 | C4BPA | up |
| ENSG00000137801 | 1.155874 | 0.005598 | THBS1 | up |
| ENSG00000154734 | 1.164505 | 0.005807 | ADAMTS1 | up |
| ENSG00000109072 | 1.977351 | 0.005912 | VTN | up |
| ENSG00000146376 | 1.171140 | 0.006384 | ARHGAP18 | up |
| ENSG00000267270 | 1.704258 | 0.006493 | PARD6G-AS1 | up |
| ENSG00000145242 | 1.654097 | 0.006686 | EPHA5 | up |
| ENSG00000100234 | 1.139636 | 0.006788 | TIMP3 | up |
| ENSG00000140403 | 2.063235 | 0.006892 | DNAJA4 | up |
| ENSG00000116701 | 1.779722 | 0.007013 | NCF2 | up |
| ENSG00000256771 | 1.288304 | 0.007174 | ZNF253 | up |
| ENSG00000249395 | 1.287375 | 0.007223 | CASC9 | up |
| ENSG00000180535 | 6.205890 | 0.007243 | BHLHA15 | up |
| ENSG00000153162 | 1.127423 | 0.007377 | BMP6 | up |
| ENSG00000111799 | 1.109504 | 0.007705 | COL12A1 | up |
| ENSG00000181690 | 1.237551 | 0.007794 | PLAG1 | up |
| ENSG00000260025 | 1.479121 | 0.007809 | AC009414.2 | up |
| ENSG00000236675 | 1.172204 | 0.007858 | MTX1P1 | up |
| ENSG00000198785 | 2.647726 | 0.007865 | GRIN3A | up |
| ENSG00000204175 | 2.647726 | 0.007865 | GPRIN2 | up |
| ENSG00000123700 | 3.276038 | 0.008390 | KCNJ2 | up |
| ENSG00000132563 | 3.276038 | 0.008390 | REEP2 | up |
| ENSG00000250722 | 3.276038 | 0.008390 | SELENOP | up |
| ENSG00000215182 | 3.276038 | 0.008390 | MUC5AC | up |
| ENSG00000183775 | 3.276038 | 0.008390 | KCTD16 | up |
| ENSG00000071242 | 3.276038 | 0.008390 | RPS6KA2 | up |
| ENSG00000132718 | 2.544721 | 0.008459 | SYT11 | up |
| ENSG00000187627 | 2.544721 | 0.008459 | RGPD1 | up |
| ENSG00000179314 | 1.674817 | 0.008735 | WSCD1 | up |
| ENSG00000241418 | 2.016141 | 0.008985 | MCRIP2P1 | up |
| ENSG00000196220 | 1.469518 | 0.009031 | SRGAP3 | up |
| ENSG00000178776 | 1.620322 | 0.009084 | C5orf46 | up |
| ENSG00000170775 | 1.258541 | 0.009209 | GPR37 | up |
| ENSG00000055813 | 1.365289 | 0.009712 | CCDC85A | up |
| ENSG00000116690 | 1.890251 | 0.009731 | PRG4 | up |
| ENSG00000175745 | 1.170295 | 0.009736 | NR2F1 | up |
| ENSG00000237412 | 1.101924 | 0.009821 | PRSS56 | up |
| ENSG00000142871 | 1.073055 | 0.010200 | CYR61 | up |
| ENSG00000271447 | 1.787565 | 0.010384 | MMP28 | up |
| ENSG00000181634 | 2.068427 | 0.010812 | TNFSF15 | up |
| ENSG00000237187 | 1.629504 | 0.011463 | NR2F1-AS1 | up |
| ENSG00000148204 | 1.629504 | 0.011463 | CRB2 | up |
| ENSG00000006016 | 1.311347 | 0.011574 | CRLF1 | up |
| ENSG00000136367 | 2.555435 | 0.011832 | ZFHX2 | up |
| ENSG00000134042 | 2.555435 | 0.011832 | MRO | up |
| ENSG00000109846 | 1.415395 | 0.012346 | CRYAB | up |
| ENSG00000267257 | 1.612268 | 0.012652 | AC105105.1 | up |
| ENSG00000111181 | 1.612268 | 0.012652 | SLC6A12 | up |
| ENSG00000111335 | 6.056057 | 0.012731 | OAS2 | up |
| ENSG00000230074 | 6.056057 | 0.012731 | AL162231.2 | up |
| ENSG00000100505 | 6.056057 | 0.012731 | TRIM9 | up |
| ENSG00000249031 | 6.056057 | 0.012731 | SUMO2P6 | up |
| ENSG00000107099 | 6.056057 | 0.012731 | DOCK8 | up |
| ENSG00000228903 | 6.056057 | 0.012731 | RASA4CP | up |
| ENSG00000279421 | 6.056057 | 0.012731 | AC067863.3 | up |
| ENSG00000228010 | 6.056057 | 0.012731 | AC073343.2 | up |
| ENSG00000147655 | 6.056057 | 0.012731 | RSPO2 | up |
| ENSG00000226686 | 6.056057 | 0.012731 | LINC01535 | up |
| ENSG00000152767 | 1.112645 | 0.013338 | FARP1 | up |
| ENSG00000205795 | 3.161817 | 0.013412 | CYS1 | up |
| ENSG00000163053 | 3.161817 | 0.013412 | SLC16A14 | up |
| ENSG00000118194 | 3.161817 | 0.013412 | TNNT2 | up |
| ENSG00000272186 | 3.161817 | 0.013412 | AP003392.6 | up |
| ENSG00000268536 | 3.161817 | 0.013412 | AC005523.1 | up |
| ENSG00000206077 | 1.462892 | 0.013710 | ZDHHC11B | up |
| ENSG00000278996 | 1.530804 | 0.014584 | FP671120.1 | up |
| ENSG00000123388 | 1.186372 | 0.014722 | HOXC11 | up |
| ENSG00000135919 | 1.010163 | 0.015350 | SERPINE2 | up |
| ENSG00000158457 | 1.118072 | 0.016065 | TSPAN33 | up |
| ENSG00000128512 | 1.024286 | 0.016377 | DOCK4 | up |
| ENSG00000164136 | 1.121756 | 0.017024 | IL15 | up |
| ENSG00000106484 | 1.245563 | 0.017036 | MEST | up |
| ENSG00000162458 | 1.029005 | 0.017271 | FBLIM1 | up |
| ENSG00000250182 | 1.551790 | 0.017539 | EEF1A1P13 | up |
| ENSG00000137752 | 2.456834 | 0.017862 | CASP1 | up |
| ENSG00000228218 | 2.456834 | 0.017862 | ATF4P3 | up |
| ENSG00000070731 | 2.456834 | 0.017862 | ST6GALNAC2 | up |
| ENSG00000258230 | 2.456834 | 0.017862 | AC063950.1 | up |
| ENSG00000168874 | 1.064961 | 0.017884 | ATOH8 | up |
| ENSG00000146166 | 1.546884 | 0.018332 | LGSN | up |
| ENSG00000172985 | 1.164609 | 0.018679 | SH3RF3 | up |
| ENSG00000164659 | 1.219364 | 0.018849 | KIAA1324L | up |
| ENSG00000151150 | 1.210855 | 0.018950 | ANK3 | up |
| ENSG00000137261 | 1.067157 | 0.018994 | KIAA0319 | up |
| ENSG00000183688 | 1.953581 | 0.019137 | RFLNB | up |
| ENSG00000124839 | 1.021377 | 0.019523 | RAB17 | up |
| ENSG00000241322 | 1.810701 | 0.019988 | CDRT1 | up |
| ENSG00000219891 | 1.698486 | 0.020624 | ZSCAN12P1 | up |
| ENSG00000177707 | 1.001534 | 0.020869 | NECTIN3 | up |
| ENSG00000188710 | 2.088641 | 0.020952 | QRFP | up |
| ENSG00000196267 | 2.088641 | 0.020952 | ZNF836 | up |
| ENSG00000173432 | 2.088641 | 0.020952 | SAA1 | up |
| ENSG00000110881 | 1.010078 | 0.021086 | ASIC1 | up |
| ENSG00000132031 | 1.576976 | 0.021444 | MATN3 | up |
| ENSG00000165887 | 1.274226 | 0.021553 | ANKRD2 | up |
| ENSG00000239636 | 3.037769 | 0.021567 | AC004865.2 | up |
| ENSG00000259656 | 3.037769 | 0.021567 | AC013452.2 | up |
| ENSG00000256249 | 3.037769 | 0.021567 | AC026333.3 | up |
| ENSG00000112902 | 1.271815 | 0.021782 | SEMA5A | up |
| ENSG00000139329 | 1.149153 | 0.021802 | LUM | up |
| ENSG00000198156 | 1.352154 | 0.022074 | NPIPB6 | up |
| ENSG00000137965 | 1.465502 | 0.022103 | IFI44 | up |
| ENSG00000186026 | 5.888840 | 0.022677 | ZNF284 | up |
| ENSG00000115290 | 5.888840 | 0.022677 | GRB14 | up |
| ENSG00000267491 | 5.888840 | 0.022677 | AC100788.1 | up |
| ENSG00000115828 | 5.888840 | 0.022677 | QPCT | up |
| ENSG00000234465 | 5.888840 | 0.022677 | PINLYP | up |
| ENSG00000147168 | 5.888840 | 0.022677 | IL2RG | up |
| ENSG00000105664 | 5.888840 | 0.022677 | COMP | up |
| ENSG00000134376 | 5.888840 | 0.022677 | CRB1 | up |
| ENSG00000100298 | 5.888840 | 0.022677 | APOBEC3H | up |
| ENSG00000179935 | 5.888840 | 0.022677 | LINC00652 | up |
| ENSG00000258365 | 5.888840 | 0.022677 | AC073655.2 | up |
| ENSG00000131484 | 5.888840 | 0.022677 | AC091132.1 | up |
| ENSG00000270021 | 5.888840 | 0.022677 | AC026691.1 | up |
| ENSG00000241527 | 5.888840 | 0.022677 | CA15P1 | up |
| ENSG00000242852 | 5.888840 | 0.022677 | ZNF709 | up |
| ENSG00000275441 | 5.888840 | 0.022677 | AC020765.2 | up |
| ENSG00000244405 | 1.019818 | 0.022755 | ETV5 | up |
| ENSG00000167562 | 1.187351 | 0.022901 | ZNF701 | up |
| ENSG00000073910 | 1.107872 | 0.023001 | FRY | up |
| ENSG00000249996 | 2.257201 | 0.023071 | AC106786.2 | up |
| ENSG00000160229 | 2.334629 | 0.023071 | ZNF66 | up |
| ENSG00000186086 | 2.257201 | 0.023071 | NBPF6 | up |
| ENSG00000214784 | 2.334629 | 0.023071 | AC010468.1 | up |
| ENSG00000232406 | 2.257201 | 0.023071 | AL121895.1 | up |
| ENSG00000152926 | 1.892538 | 0.025475 | ZNF117 | up |
| ENSG00000178665 | 1.063809 | 0.026102 | ZNF713 | up |
| ENSG00000112320 | 1.754421 | 0.026105 | SOBP | up |
| ENSG00000260075 | 1.559084 | 0.026720 | AC217777.1 | up |
| ENSG00000130513 | 1.005293 | 0.026971 | GDF15 | up |
| ENSG00000169129 | 2.350997 | 0.027043 | AFAP1L2 | up |
| ENSG00000272979 | 2.350997 | 0.027043 | AC093388.1 | up |
| ENSG00000226763 | 2.350997 | 0.027043 | SRRM5 | up |
| ENSG00000233327 | 2.350997 | 0.027043 | USP32P2 | up |
| ENSG00000273010 | 2.350997 | 0.027043 | AL360270.3 | up |
| ENSG00000248690 | 2.350997 | 0.027043 | HAS2-AS1 | up |
| ENSG00000145506 | 1.008991 | 0.027872 | NKD2 | up |
| ENSG00000086619 | 1.200424 | 0.028510 | ERO1B | up |
| ENSG00000153993 | 1.030088 | 0.029306 | SEMA3D | up |
| ENSG00000151320 | 1.151848 | 0.029317 | AKAP6 | up |
| ENSG00000234817 | 1.267191 | 0.029543 | AL136309.2 | up |
| ENSG00000101955 | 1.265284 | 0.029914 | SRPX | up |
| ENSG00000182308 | 1.293425 | 0.030299 | DCAF4L1 | up |
| ENSG00000272574 | 1.996350 | 0.030348 | AL596325.2 | up |
| ENSG00000241889 | 1.996350 | 0.030348 | AC079944.2 | up |
| ENSG00000108452 | 1.996350 | 0.030348 | ZNF29P | up |
| ENSG00000255650 | 1.996350 | 0.030348 | FAM222A-AS1 | up |
| ENSG00000154556 | 1.149708 | 0.031014 | SORBS2 | up |
| ENSG00000136943 | 1.053630 | 0.031199 | CTSV | up |
| ENSG00000253217 | 1.375263 | 0.032779 | AP001574.1 | up |
| ENSG00000105650 | 1.426757 | 0.033174 | PDE4C | up |
| ENSG00000205085 | 1.426757 | 0.033174 | FAM71F2 | up |
| ENSG00000128271 | 2.006189 | 0.033376 | ADORA2A | up |
| ENSG00000206754 | 2.006189 | 0.033376 | SNORD101 | up |
| ENSG00000153294 | 2.006189 | 0.033376 | ADGRF4 | up |
| ENSG00000143786 | 1.009423 | 0.033671 | CNIH3 | up |
| ENSG00000124203 | 1.508701 | 0.033832 | ZNF831 | up |
| ENSG00000228412 | 1.695855 | 0.034088 | AL022068.1 | up |
| ENSG00000226258 | 1.236219 | 0.034843 | GRM7-AS3 | up |
| ENSG00000123977 | 2.902044 | 0.034867 | DAW1 | up |
| ENSG00000200816 | 2.902044 | 0.034867 | SNORA38 | up |
| ENSG00000142449 | 2.902044 | 0.034867 | FBN3 | up |
| ENSG00000274677 | 2.902044 | 0.034867 | AC040169.3 | up |
| ENSG00000252174 | 2.902044 | 0.034867 | RNU7-18P | up |
| ENSG00000257818 | 2.902044 | 0.034867 | C1GALT1P1 | up |
| ENSG00000280039 | 2.902044 | 0.034867 | RN7SKP23 | up |
| ENSG00000258593 | 2.902044 | 0.034867 | AL583810.1 | up |
| ENSG00000170629 | 1.263138 | 0.035482 | DPY19L2P2 | up |
| ENSG00000279148 | 1.263138 | 0.035482 | AC126474.1 | up |
| ENSG00000150636 | 1.293027 | 0.036157 | CCDC102B | up |
| ENSG00000180998 | 1.113444 | 0.036411 | GPR137C | up |
| ENSG00000225614 | 1.169389 | 0.038025 | ZNF469 | up |
| ENSG00000187398 | 1.189660 | 0.038818 | LUZP2 | up |
| ENSG00000112183 | 1.071523 | 0.039100 | RBM24 | up |
| ENSG00000128573 | 1.291437 | 0.039238 | FOXP2 | up |
| ENSG00000105750 | 1.067447 | 0.039577 | ZNF85 | up |
| ENSG00000184923 | 1.206555 | 0.040568 | NUTM2A | up |
| ENSG00000144218 | 5.481896 | 0.040965 | AFF3 | up |
| ENSG00000162817 | 5.699669 | 0.040965 | C1orf115 | up |
| ENSG00000121406 | 5.699669 | 0.040965 | ZNF549 | up |
| ENSG00000153208 | 5.481896 | 0.040965 | MERTK | up |
| ENSG00000140511 | 5.699669 | 0.040965 | HAPLN3 | up |
| ENSG00000088992 | 5.699669 | 0.040965 | TESC | up |
| ENSG00000112599 | 5.699669 | 0.040965 | GUCA1B | up |
| ENSG00000225556 | 5.481896 | 0.040965 | C2CD4D | up |
| ENSG00000199719 | 5.481896 | 0.040965 | RN7SKP74 | up |
| ENSG00000229839 | 5.481896 | 0.040965 | AC018462.1 | up |
| ENSG00000231290 | 5.481896 | 0.040965 | APCDD1L-AS1 | up |
| ENSG00000279390 | 5.699669 | 0.040965 | AF127577.6 | up |
| ENSG00000242889 | 5.699669 | 0.040965 | RN7SL449P | up |
| ENSG00000128833 | 5.481896 | 0.040965 | MYO5C | up |
| ENSG00000280379 | 5.481896 | 0.040965 | AP003072.5 | up |
| ENSG00000224550 | 5.699669 | 0.040965 | AC114491.1 | up |
| ENSG00000252916 | 5.481896 | 0.040965 | RNU6-762P | up |
| ENSG00000254484 | 5.481896 | 0.040965 | AP002336.1 | up |
| ENSG00000203435 | 5.481896 | 0.040965 | E2F3P2 | up |
| ENSG00000256312 | 5.481896 | 0.040965 | AC138466.1 | up |
| ENSG00000269054 | 5.481896 | 0.040965 | AC012313.6 | up |
| ENSG00000229896 | 5.699669 | 0.040965 | AL157373.2 | up |
| ENSG00000236051 | 5.699669 | 0.040965 | MYCBP2-AS1 | up |
| ENSG00000248663 | 5.699669 | 0.040965 | LINC00992 | up |
| ENSG00000257802 | 5.699669 | 0.040965 | AC089984.2 | up |
| ENSG00000233791 | 5.699669 | 0.040965 | LINC01136 | up |
| ENSG00000277481 | 5.481896 | 0.040965 | PKD1L3 | up |
| ENSG00000251396 | 5.699669 | 0.040965 | LINC01301 | up |
| ENSG00000231344 | 5.699669 | 0.040965 | AL020997.1 | up |
| ENSG00000146005 | 5.481896 | 0.040965 | PSD2 | up |
| ENSG00000255910 | 5.481896 | 0.040965 | AC024901.1 | up |
| ENSG00000272563 | 5.481896 | 0.040965 | AC016745.2 | up |
| ENSG00000231333 | 5.699669 | 0.040965 | RPL34P6 | up |
| ENSG00000112276 | 5.699669 | 0.040965 | BVES | up |
| ENSG00000271817 | 5.481896 | 0.040965 | U3 | up |
| ENSG00000252122 | 5.699669 | 0.040965 | SNORA76 | up |
| ENSG00000277382 | 5.481896 | 0.040965 | AC005837.3 | up |
| ENSG00000070526 | 5.699669 | 0.040965 | ST6GALNAC1 | up |
| ENSG00000232527 | 5.481896 | 0.040965 | AC245595.1 | up |
| ENSG00000214433 | 5.699669 | 0.040965 | GOLGA2P8 | up |
| ENSG00000258952 | 5.481896 | 0.040965 | SALRNA1 | up |
| ENSG00000277327 | 5.481896 | 0.040965 | SPDYE20P | up |
| ENSG00000167754 | 5.481896 | 0.040965 | KLK5 | up |
| ENSG00000163449 | 5.481896 | 0.040965 | TMEM169 | up |
| ENSG00000225272 | 5.481896 | 0.040965 | AL451074.3 | up |
| ENSG00000261581 | 5.481896 | 0.040965 | HERC2P11 | up |
| ENSG00000257845 | 5.481896 | 0.040965 | LINC02294 | up |
| ENSG00000271490 | 5.481896 | 0.040965 | AC013244.2 | up |
| ENSG00000241735 | 5.481896 | 0.040965 | FABP5P3 | up |
| ENSG00000225364 | 5.481896 | 0.040965 | ATP6V0E1P1 | up |
| ENSG00000266242 | 5.699669 | 0.040965 | GRAMD4P7 | up |
| ENSG00000249006 | 5.481896 | 0.040965 | AL136360.1 | up |
| ENSG00000230162 | 5.481896 | 0.040965 | CT45A11P | up |
| ENSG00000232057 | 5.481896 | 0.040965 | AC093390.1 | up |
| ENSG00000276083 | 5.481896 | 0.040965 | MIR7976 | up |
| ENSG00000267106 | 1.382550 | 0.040994 | ZNF561-AS1 | up |
| ENSG00000261442 | 2.236776 | 0.041032 | AC023830.1 | up |
| ENSG00000248874 | 2.236776 | 0.041032 | C5orf17 | up |
| ENSG00000174990 | 2.236776 | 0.041032 | CA5A | up |
| ENSG00000171617 | 1.085679 | 0.041364 | ENC1 | up |
| ENSG00000169946 | 1.232203 | 0.041535 | ZFPM2 | up |
| ENSG00000228212 | 1.440070 | 0.041904 | OFD1P17 | up |
| ENSG00000253598 | 1.125315 | 0.042612 | SLC10A5 | up |
| ENSG00000272084 | 1.456494 | 0.042814 | AL137127.1 | up |
| ENSG00000176884 | 1.897749 | 0.043991 | GRIN1 | up |
| ENSG00000261740 | 1.897749 | 0.043991 | BOLA2-SMG1P6 | up |
| ENSG00000212327 | 1.897749 | 0.043991 | RNU6-882P | up |
| ENSG00000272656 | 1.897749 | 0.043991 | AC024933.1 | up |
| ENSG00000235837 | 1.897749 | 0.043991 | AC073333.1 | up |
| ENSG00000105988 | 1.897749 | 0.043991 | NHP2P1 | up |
| ENSG00000188263 | 1.634812 | 0.044489 | IL17REL | up |
| ENSG00000185634 | 1.634812 | 0.044489 | SHC4 | up |
| ENSG00000279413 | 1.162251 | 0.044744 | AC112497.1 | up |
| ENSG00000284428 | 1.117834 | 0.045117 | AC092329.4 | up |
| ENSG00000168016 | 1.932706 | 0.045436 | TRANK1 | up |
| ENSG00000259863 | 1.932706 | 0.045436 | SH3RF3-AS1 | up |
| ENSG00000125246 | 1.330817 | 0.046163 | CLYBL | up |
| ENSG00000183671 | 1.089533 | 0.048420 | GPR1 | up |
| ENSG00000214076 | 1.099384 | 0.048686 | CPSF1P1 | up |
| ENSG00000054690 | 1.290716 | 0.049028 | PLEKHH1 | up |
| ENSG00000105141 | 6.011343 | 2.55E-15 | CASP14 | down |
| ENSG00000249839 | 3.084592 | 5.46E-09 | AC011330.1 | down |
| ENSG00000263934 | 3.681136 | 1.38E-08 | SNORD3A | down |
| ENSG00000188985 | 2.309942 | 2.65E-07 | DHFRP1 | down |
| ENSG00000270276 | 3.019853 | 7.58E-07 | HIST2H4B | down |
| ENSG00000130487 | 4.194797 | 5.52E-06 | KLHDC7B | down |
| ENSG00000135744 | 3.098950 | 2.41E-05 | AGT | down |
| ENSG00000138271 | 2.214663 | 2.82E-05 | GPR87 | down |
| ENSG00000214049 | 2.254979 | 7.67E-05 | UCA1 | down |
| ENSG00000204580 | 1.645808 | 0.000102 | DDR1 | down |
| ENSG00000165029 | 1.658852 | 0.000137 | ABCA1 | down |
| ENSG00000186847 | 2.663991 | 0.000138 | KRT14 | down |
| ENSG00000228742 | 2.943514 | 0.000215 | AC002384.1 | down |
| ENSG00000008323 | 1.912962 | 0.000241 | PLEKHG6 | down |
| ENSG00000147100 | 1.631156 | 0.000315 | SLC16A2 | down |
| ENSG00000134769 | 1.578935 | 0.000345 | DTNA | down |
| ENSG00000143365 | 1.709493 | 0.000452 | RORC | down |
| ENSG00000224389 | 1.593356 | 0.000642 | C4B | down |
| ENSG00000253671 | 1.983520 | 0.000708 | AC027117.1 | down |
| ENSG00000258301 | 3.446184 | 0.000740 | VASH1-AS1 | down |
| ENSG00000243649 | 1.538874 | 0.000764 | CFB | down |
| ENSG00000168785 | 2.169716 | 0.000786 | TSPAN5 | down |
| ENSG00000199846 | 6.748266 | 0.000858 | RNU1-72P | down |
| ENSG00000205622 | 2.574861 | 0.000872 | AP001043.1 | down |
| ENSG00000163995 | 1.731589 | 0.000959 | ABLIM2 | down |
| ENSG00000115255 | 1.558050 | 0.001047 | REEP6 | down |
| ENSG00000273033 | 1.840630 | 0.001055 | LINC02035 | down |
| ENSG00000233783 | 2.814926 | 0.001085 | AP001442.1 | down |
| ENSG00000122547 | 1.891229 | 0.001170 | EEPD1 | down |
| ENSG00000273343 | 2.428820 | 0.001185 | AC007663.4 | down |
| ENSG00000135678 | 1.419309 | 0.001213 | CPM | down |
| ENSG00000221930 | 1.596065 | 0.001576 | FAM45BP | down |
| ENSG00000179913 | 1.453964 | 0.001661 | B3GNT3 | down |
| ENSG00000181790 | 1.667347 | 0.001701 | ADGRB1 | down |
| ENSG00000273117 | 1.663172 | 0.001716 | AC144652.1 | down |
| ENSG00000278817 | 1.640952 | 0.001728 | AC007325.4 | down |
| ENSG00000107159 | 1.342175 | 0.001733 | CA9 | down |
| ENSG00000198417 | 1.537401 | 0.001749 | MT1F | down |
| ENSG00000205583 | 1.473381 | 0.001909 | STAG3L1 | down |
| ENSG00000151176 | 1.300252 | 0.002031 | PLBD2 | down |
| ENSG00000205809 | 1.438921 | 0.002099 | KLRC2 | down |
| ENSG00000163885 | 6.487913 | 0.002435 | CFAP100 | down |
| ENSG00000134061 | 1.795537 | 0.002641 | CD180 | down |
| ENSG00000071282 | 1.456191 | 0.002795 | LMCD1 | down |
| ENSG00000166592 | 1.324238 | 0.002822 | RRAD | down |
| ENSG00000228137 | 1.790084 | 0.002910 | AP001469.2 | down |
| ENSG00000128578 | 1.258863 | 0.003057 | STRIP2 | down |
| ENSG00000126217 | 1.349725 | 0.003172 | MCF2L | down |
| ENSG00000230795 | 3.812775 | 0.003346 | HLA-K | down |
| ENSG00000202441 | 3.812775 | 0.003346 | RNY4P10 | down |
| ENSG00000183542 | 2.415763 | 0.003385 | KLRC4 | down |
| ENSG00000230013 | 2.415763 | 0.003385 | AL359182.2 | down |
| ENSG00000284194 | 2.415763 | 0.003385 | SCO2 | down |
| ENSG00000153531 | 1.289170 | 0.003390 | ADPRHL1 | down |
| ENSG00000080031 | 3.199850 | 0.003515 | PTPRH | down |
| ENSG00000157613 | 1.289306 | 0.003527 | CREB3L1 | down |
| ENSG00000165181 | 1.279627 | 0.003835 | C9orf84 | down |
| ENSG00000273820 | 1.783776 | 0.003981 | USP27X | down |
| ENSG00000258171 | 6.337694 | 0.004174 | LINC02412 | down |
| ENSG00000138829 | 1.211922 | 0.004264 | FBN2 | down |
| ENSG00000283646 | 1.451973 | 0.004269 | LINC02009 | down |
| ENSG00000128203 | 2.254550 | 0.004369 | ASPHD2 | down |
| ENSG00000047457 | 1.503660 | 0.004484 | CP | down |
| ENSG00000159403 | 1.215401 | 0.004873 | C1R | down |
| ENSG00000117983 | 1.744369 | 0.004914 | MUC5B | down |
| ENSG00000154319 | 1.237177 | 0.004959 | FAM167A | down |
| ENSG00000213846 | 2.021876 | 0.005291 | AC098614.1 | down |
| ENSG00000143847 | 1.214399 | 0.005359 | PPFIA4 | down |
| ENSG00000170989 | 1.361299 | 0.005432 | S1PR1 | down |
| ENSG00000133739 | 1.631737 | 0.005639 | LRRCC1 | down |
| ENSG00000164251 | 1.198406 | 0.005804 | F2RL1 | down |
| ENSG00000280587 | 2.180975 | 0.006073 | FP700125.1 | down |
| ENSG00000232456 | 2.180975 | 0.006073 | AL355994.2 | down |
| ENSG00000237596 | 2.041871 | 0.006128 | AL138828.1 | down |
| ENSG00000105048 | 1.341933 | 0.006181 | TNNT1 | down |
| ENSG00000176087 | 1.140409 | 0.006334 | SLC35A4 | down |
| ENSG00000110876 | 1.283794 | 0.006451 | SELPLG | down |
| ENSG00000142619 | 1.677938 | 0.006493 | PADI3 | down |
| ENSG00000255159 | 2.333829 | 0.006966 | AC026894.1 | down |
| ENSG00000130720 | 1.137383 | 0.007042 | FIBCD1 | down |
| ENSG00000060656 | 1.167762 | 0.007147 | PTPRU | down |
| ENSG00000174521 | 6.169997 | 0.007243 | TTC9B | down |
| ENSG00000130822 | 6.169997 | 0.007243 | PNCK | down |
| ENSG00000155714 | 6.169997 | 0.007243 | PDZD9 | down |
| ENSG00000215893 | 6.169997 | 0.007243 | RPL23AP17 | down |
| ENSG00000201813 | 6.169997 | 0.007243 | RNU6-915P | down |
| ENSG00000128310 | 6.169997 | 0.007243 | GALR3 | down |
| ENSG00000196302 | 6.169997 | 0.007243 | AC146944.1 | down |
| ENSG00000277476 | 1.414814 | 0.007431 | AC005332.8 | down |
| ENSG00000186907 | 1.776393 | 0.007499 | RTN4RL2 | down |
| ENSG00000154237 | 1.240038 | 0.007578 | LRRK1 | down |
| ENSG00000184785 | 1.129405 | 0.007973 | SMIM10 | down |
| ENSG00000077238 | 1.139307 | 0.008140 | IL4R | down |
| ENSG00000015568 | 1.297543 | 0.008191 | RGPD5 | down |
| ENSG00000197261 | 1.339634 | 0.008226 | C6orf141 | down |
| ENSG00000246851 | 3.574019 | 0.008390 | AL157938.3 | down |
| ENSG00000243738 | 3.574019 | 0.008390 | RN7SL181P | down |
| ENSG00000265739 | 3.574019 | 0.008390 | AC104984.3 | down |
| ENSG00000256982 | 3.574019 | 0.008390 | AC135782.1 | down |
| ENSG00000074410 | 1.091907 | 0.008898 | CA12 | down |
| ENSG00000172061 | 1.547977 | 0.009084 | LRRC15 | down |
| ENSG00000020633 | 1.534686 | 0.009406 | RUNX3 | down |
| ENSG00000178695 | 1.190243 | 0.009520 | KCTD12 | down |
| ENSG00000188505 | 1.369209 | 0.009559 | NCCRP1 | down |
| ENSG00000196979 | 1.522679 | 0.009704 | AL360004.1 | down |
| ENSG00000232748 | 1.997958 | 0.009733 | AC135050.1 | down |
| ENSG00000249012 | 1.997958 | 0.009733 | AC104819.1 | down |
| ENSG00000141750 | 1.162558 | 0.009752 | STAC2 | down |
| ENSG00000166002 | 1.321766 | 0.009997 | SMCO4 | down |
| ENSG00000117477 | 2.246960 | 0.010034 | CCDC181 | down |
| ENSG00000220920 | 2.246960 | 0.010034 | AL023807.1 | down |
| ENSG00000087303 | 1.071483 | 0.010583 | NID2 | down |
| ENSG00000099953 | 1.395904 | 0.010695 | MMP11 | down |
| ENSG00000168298 | 1.916953 | 0.010812 | HIST1H1E | down |
| ENSG00000186918 | 1.111318 | 0.011150 | ZNF395 | down |
| ENSG00000257475 | 1.500828 | 0.011159 | AC068888.2 | down |
| ENSG00000184451 | 1.252769 | 0.011201 | CCR10 | down |
| ENSG00000179954 | 1.852700 | 0.011724 | SSC5D | down |
| ENSG00000234945 | 1.852700 | 0.011724 | GTF3C2-AS1 | down |
| ENSG00000277053 | 1.067737 | 0.011742 | GTF2IP1 | down |
| ENSG00000197632 | 2.902616 | 0.011832 | SERPINB2 | down |
| ENSG00000174370 | 1.145126 | 0.012395 | C11orf45 | down |
| ENSG00000171060 | 1.487046 | 0.012652 | C8orf74 | down |
| ENSG00000150764 | 1.060294 | 0.012685 | DIXDC1 | down |
| ENSG00000171450 | 5.980212 | 0.012731 | CDK5R2 | down |
| ENSG00000049283 | 5.980212 | 0.012731 | EPN3 | down |
| ENSG00000232536 | 5.980212 | 0.012731 | AL365436.2 | down |
| ENSG00000225131 | 5.980212 | 0.012731 | PSME2P2 | down |
| ENSG00000283973 | 5.980212 | 0.012731 | AC099795.1 | down |
| ENSG00000269066 | 5.980212 | 0.012731 | AC020908.3 | down |
| ENSG00000270174 | 5.980212 | 0.012731 | AL022097.1 | down |
| ENSG00000270324 | 5.980212 | 0.012731 | AC005972.2 | down |
| ENSG00000130775 | 1.924382 | 0.013231 | THEMIS2 | down |
| ENSG00000254369 | 1.924382 | 0.013231 | HOXA-AS3 | down |
| ENSG00000183186 | 3.437978 | 0.013412 | C2CD4C | down |
| ENSG00000156097 | 3.437978 | 0.013412 | GPR61 | down |
| ENSG00000237331 | 3.437978 | 0.013412 | XIAP-AS1 | down |
| ENSG00000228120 | 3.437978 | 0.013412 | AP001631.1 | down |
| ENSG00000167680 | 1.086239 | 0.013563 | SEMA6B | down |
| ENSG00000235245 | 1.188656 | 0.013607 | AL360181.2 | down |
| ENSG00000235831 | 1.671780 | 0.013705 | BHLHE40-AS1 | down |
| ENSG00000220785 | 1.088279 | 0.013795 | MTMR9LP | down |
| ENSG00000197557 | 1.405472 | 0.014090 | TTC30A | down |
| ENSG00000230454 | 1.850189 | 0.014380 | U73166.1 | down |
| ENSG00000163349 | 1.027749 | 0.014402 | HIPK1 | down |
| ENSG00000244218 | 2.553686 | 0.014485 | RN7SL81P | down |
| ENSG00000241217 | 2.553686 | 0.014485 | RN7SL809P | down |
| ENSG00000261744 | 2.553686 | 0.014485 | AC116552.1 | down |
| ENSG00000253944 | 2.154523 | 0.014485 | AC027117.2 | down |
| ENSG00000170379 | 1.499417 | 0.014931 | TCAF2 | down |
| ENSG00000266777 | 1.499417 | 0.014931 | AC090616.6 | down |
| ENSG00000171236 | 1.499417 | 0.014931 | LRG1 | down |
| ENSG00000259623 | 1.090440 | 0.014936 | AC125257.1 | down |
| ENSG00000180610 | 1.791593 | 0.015305 | ZBTB12BP | down |
| ENSG00000149418 | 1.153596 | 0.015902 | ST14 | down |
| ENSG00000183054 | 1.423401 | 0.015903 | RGPD6 | down |
| ENSG00000272523 | 1.744107 | 0.016052 | LINC01023 | down |
| ENSG00000256817 | 1.410906 | 0.016216 | TPT1P12 | down |
| ENSG00000129173 | 1.053812 | 0.016472 | E2F8 | down |
| ENSG00000179242 | 2.021510 | 0.016493 | CDH4 | down |
| ENSG00000133020 | 2.021510 | 0.016493 | MYH8 | down |
| ENSG00000054356 | 2.021510 | 0.016493 | PTPRN | down |
| ENSG00000007944 | 1.021380 | 0.016634 | MYLIP | down |
| ENSG00000169884 | 1.042050 | 0.016813 | WNT10B | down |
| ENSG00000261730 | 1.210644 | 0.017108 | AL034346.1 | down |
| ENSG00000197249 | 2.788172 | 0.017862 | SERPINA1 | down |
| ENSG00000201616 | 2.788172 | 0.017862 | RNU1-91P | down |
| ENSG00000261527 | 2.788172 | 0.017862 | AC026464.5 | down |
| ENSG00000196415 | 1.846852 | 0.018003 | PRTN3 | down |
| ENSG00000162894 | 1.447639 | 0.018332 | FCMR | down |
| ENSG00000197822 | 1.394210 | 0.018640 | OCLN | down |
| ENSG00000268879 | 1.091640 | 0.018824 | IGFL1P1 | down |
| ENSG00000248100 | 1.780184 | 0.019137 | AC087257.1 | down |
| ENSG00000143515 | 1.076867 | 0.019372 | ATP8B2 | down |
| ENSG00000165507 | 1.685483 | 0.020624 | C10orf10 | down |
| ENSG00000162630 | 2.055755 | 0.020952 | B3GALT2 | down |
| ENSG00000125508 | 2.454918 | 0.020952 | SRMS | down |
| ENSG00000011590 | 2.055755 | 0.020952 | ZBTB32 | down |
| ENSG00000236519 | 1.650608 | 0.021096 | LINC01424 | down |
| ENSG00000259475 | 1.569100 | 0.021444 | AC036108.1 | down |
| ENSG00000234076 | 3.287759 | 0.021567 | TPRG1-AS1 | down |
| ENSG00000227331 | 3.287759 | 0.021567 | AC005042.1 | down |
| ENSG00000251867 | 3.287759 | 0.021567 | AC009812.1 | down |
| ENSG00000262380 | 3.287759 | 0.021567 | AC026401.2 | down |
| ENSG00000231703 | 3.287759 | 0.021567 | AL354993.1 | down |
| ENSG00000175264 | 3.287759 | 0.021567 | CHST1 | down |
| ENSG00000264424 | 1.547732 | 0.021695 | MYH4 | down |
| ENSG00000186056 | 1.529366 | 0.021872 | MATN1-AS1 | down |
| ENSG00000272473 | 1.423529 | 0.021872 | AC006273.1 | down |
| ENSG00000220472 | 1.414809 | 0.021991 | AL139095.2 | down |
| ENSG00000227038 | 1.414809 | 0.021991 | GTF2IP7 | down |
| ENSG00000107736 | 1.113639 | 0.023239 | CDH23 | down |
| ENSG00000104043 | 1.001099 | 0.023256 | ATP8B4 | down |
| ENSG00000185565 | 1.215008 | 0.023741 | LSAMP | down |
| ENSG00000176490 | 1.764918 | 0.024511 | DIRAS1 | down |
| ENSG00000187714 | 1.142156 | 0.024694 | SLC18A3 | down |
| ENSG00000179583 | 1.142156 | 0.024694 | CIITA | down |
| ENSG00000182463 | 1.024632 | 0.025458 | TSHZ2 | down |
| ENSG00000280160 | 1.706609 | 0.025475 | AC135050.7 | down |
| ENSG00000223703 | 1.706609 | 0.025475 | AC027612.1 | down |
| ENSG00000204044 | 1.661018 | 0.026105 | SLC12A5-AS1 | down |
| ENSG00000160801 | 1.661018 | 0.026105 | PTH1R | down |
| ENSG00000263050 | 1.624375 | 0.026498 | AC090617.6 | down |
| ENSG00000207146 | 1.624375 | 0.026498 | Y_RNA | down |
| ENSG00000146904 | 1.624375 | 0.026498 | EPHA1 | down |
| ENSG00000227355 | 1.365442 | 0.026552 | AL359644.1 | down |
| ENSG00000268262 | 2.663860 | 0.027043 | AC011445.1 | down |
| ENSG00000123500 | 2.663860 | 0.027043 | COL10A1 | down |
| ENSG00000232259 | 2.663860 | 0.027043 | AL158166.2 | down |
| ENSG00000258352 | 2.663860 | 0.027043 | AC119044.1 | down |
| ENSG00000278899 | 2.663860 | 0.027043 | AL358852.1 | down |
| ENSG00000218073 | 2.663860 | 0.027043 | AL021407.3 | down |
| ENSG00000109625 | 1.130844 | 0.027814 | CPZ | down |
| ENSG00000049089 | 1.191820 | 0.028841 | COL9A2 | down |
| ENSG00000245648 | 1.280477 | 0.029185 | AC022075.1 | down |
| ENSG00000182584 | 1.079210 | 0.029478 | ACTL10 | down |
| ENSG00000279722 | 1.949726 | 0.030348 | AC007342.7 | down |
| ENSG00000199133 | 1.949726 | 0.030348 | MIRLET7D | down |
| ENSG00000255568 | 1.949726 | 0.030348 | BRWD1-AS2 | down |
| ENSG00000101197 | 1.012807 | 0.031656 | BIRC7 | down |
| ENSG00000171798 | 1.322517 | 0.031942 | KNDC1 | down |
| ENSG00000283031 | 1.842204 | 0.032292 | AC009242.1 | down |
| ENSG00000283696 | 1.842204 | 0.032292 | AL592295.4 | down |
| ENSG00000153012 | 1.842204 | 0.032292 | LGI2 | down |
| ENSG00000136944 | 1.514883 | 0.033533 | LMX1B | down |
| ENSG00000177679 | 1.535650 | 0.033832 | SRRM3 | down |
| ENSG00000258130 | 1.629079 | 0.033910 | AC106782.1 | down |
| ENSG00000240303 | 1.629079 | 0.033910 | ACAD11 | down |
| ENSG00000226167 | 1.629079 | 0.033910 | AP4B1-AS1 | down |
| ENSG00000259155 | 1.629079 | 0.033910 | AL591767.3 | down |
| ENSG00000071575 | 1.052312 | 0.034003 | TRIB2 | down |
| ENSG00000108924 | 1.560565 | 0.034035 | HLF | down |
| ENSG00000267475 | 1.591013 | 0.034088 | AC008736.1 | down |
| ENSG00000176092 | 1.084914 | 0.034239 | CRYBG2 | down |
| ENSG00000225791 | 1.156368 | 0.034718 | TRAM2-AS1 | down |
| ENSG00000197635 | 3.120062 | 0.034867 | DPP4 | down |
| ENSG00000196684 | 3.120062 | 0.034867 | HSH2D | down |
| ENSG00000261737 | 3.120062 | 0.034867 | AL049597.2 | down |
| ENSG00000263370 | 3.120062 | 0.034867 | AC104564.2 | down |
| ENSG00000145451 | 3.120062 | 0.034867 | GLRA3 | down |
| ENSG00000270760 | 3.120062 | 0.034867 | AD001527.1 | down |
| ENSG00000227170 | 3.120062 | 0.034867 | AF178030.1 | down |
| ENSG00000200120 | 3.120062 | 0.034867 | Y_RNA | down |
| ENSG00000251175 | 3.120062 | 0.034867 | AC008243.1 | down |
| ENSG00000166736 | 3.120062 | 0.034867 | HTR3A | down |
| ENSG00000223486 | 3.120062 | 0.034867 | AC092198.1 | down |
| ENSG00000100302 | 1.243300 | 0.035482 | RASD2 | down |
| ENSG00000122592 | 1.043785 | 0.036188 | HOXA7 | down |
| ENSG00000233554 | 1.279079 | 0.036870 | B4GALT1-AS1 | down |
| ENSG00000179399 | 1.135539 | 0.037281 | GPC5 | down |
| ENSG00000205810 | 1.213309 | 0.039664 | KLRC3 | down |
| ENSG00000275708 | 1.124301 | 0.039916 | MIR3648-1 | down |
| ENSG00000110427 | 1.277247 | 0.040101 | KIAA1549L | down |
| ENSG00000171217 | 1.277247 | 0.040101 | CLDN20 | down |
| ENSG00000147082 | 5.761623 | 0.040965 | CCNB3 | down |
| ENSG00000250900 | 5.761623 | 0.040965 | AC008443.6 | down |
| ENSG00000198400 | 5.761623 | 0.040965 | NTRK1 | down |
| ENSG00000248909 | 5.761623 | 0.040965 | HMGB1P21 | down |
| ENSG00000226738 | 5.761623 | 0.040965 | U62317.1 | down |
| ENSG00000246145 | 5.761623 | 0.040965 | RRS1-AS1 | down |
| ENSG00000236123 | 5.761623 | 0.040965 | CEACAMP11 | down |
| ENSG00000233296 | 5.761623 | 0.040965 | AC092159.2 | down |
| ENSG00000214832 | 5.761623 | 0.040965 | UPF3AP2 | down |
| ENSG00000238005 | 5.761623 | 0.040965 | AL391832.2 | down |
| ENSG00000267959 | 5.761623 | 0.040965 | MIR3188 | down |
| ENSG00000140932 | 5.761623 | 0.040965 | CMTM2 | down |
| ENSG00000273350 | 5.761623 | 0.040965 | AC004832.5 | down |
| ENSG00000271367 | 5.761623 | 0.040965 | AL034374.2 | down |
| ENSG00000280321 | 5.761623 | 0.040965 | AC129502.1 | down |
| ENSG00000259577 | 5.761623 | 0.040965 | CERNA1 | down |
| ENSG00000250061 | 5.761623 | 0.040965 | AC091976.1 | down |
| ENSG00000267174 | 5.761623 | 0.040965 | AC011472.2 | down |
| ENSG00000225215 | 5.761623 | 0.040965 | SMARCE1P1 | down |
| ENSG00000236327 | 5.761623 | 0.040965 | AL831737.1 | down |
| ENSG00000280333 | 5.761623 | 0.040965 | AC090617.10 | down |
| ENSG00000265291 | 5.761623 | 0.040965 | MIR4710 | down |
| ENSG00000253430 | 5.761623 | 0.040965 | AC011726.2 | down |
| ENSG00000250490 | 5.761623 | 0.040965 | LINC02145 | down |
| ENSG00000181781 | 5.761623 | 0.040965 | ODF3L2 | down |
| ENSG00000260860 | 5.761623 | 0.040965 | AC126773.3 | down |
| ENSG00000252552 | 5.761623 | 0.040965 | RNU6-307P | down |
| ENSG00000206887 | 5.761623 | 0.040965 | RNU6-1008P | down |
| ENSG00000228540 | 5.761623 | 0.040965 | AC073326.1 | down |
| ENSG00000235314 | 2.527819 | 0.041032 | LINC00957 | down |
| ENSG00000126262 | 2.527819 | 0.041032 | FFAR2 | down |
| ENSG00000248641 | 2.527819 | 0.041032 | HMGA1P2 | down |
| ENSG00000261714 | 2.527819 | 0.041032 | AC105137.2 | down |
| ENSG00000276980 | 1.205934 | 0.041535 | AC008760.2 | down |
| ENSG00000172638 | 1.458548 | 0.041904 | EFEMP2 | down |
| ENSG00000179772 | 1.458548 | 0.041904 | FOXS1 | down |
| ENSG00000169169 | 1.002289 | 0.043397 | CPT1C | down |
| ENSG00000225506 | 1.493801 | 0.043693 | CYP4A22-AS1 | down |
| ENSG00000268366 | 1.493801 | 0.043693 | AC010271.1 | down |
| ENSG00000249375 | 1.493801 | 0.043693 | CASC11 | down |
| ENSG00000124134 | 2.234444 | 0.043991 | KCNS1 | down |
| ENSG00000280007 | 2.234444 | 0.043991 | AC008079.2 | down |
| ENSG00000160471 | 2.234444 | 0.043991 | COX6B2 | down |
| ENSG00000146469 | 2.234444 | 0.043991 | VIP | down |
| ENSG00000242602 | 1.835281 | 0.043991 | AC008953.1 | down |
| ENSG00000279977 | 2.234444 | 0.043991 | AC008764.10 | down |
| ENSG00000226334 | 2.234444 | 0.043991 | AL359182.1 | down |
| ENSG00000232412 | 1.835281 | 0.043991 | AL121601.1 | down |
| ENSG00000106809 | 1.517438 | 0.044489 | OGN | down |
| ENSG00000188649 | 1.517438 | 0.044489 | CC2D2B | down |
| ENSG00000213937 | 1.517438 | 0.044489 | CLDN9 | down |
| ENSG00000184489 | 1.096348 | 0.045117 | PTP4A3 | down |
| ENSG00000272155 | 1.547145 | 0.045117 | AC055822.1 | down |
| ENSG00000239556 | 1.743436 | 0.045202 | AC004951.2 | down |
| ENSG00000267838 | 1.743436 | 0.045202 | AC245884.8 | down |
| ENSG00000172296 | 1.678049 | 0.045436 | SPTLC3 | down |
| ENSG00000279696 | 1.678049 | 0.045436 | AP001273.1 | down |
| ENSG00000104826 | 1.678049 | 0.045436 | LHB | down |
| ENSG00000176678 | 1.167575 | 0.048597 | FOXL1 | down |

| **Supplementary Table S5. Proteins bind with DDR1 identified by IP-MS in SK-Hep1 cells.** | | | | | | | |
| --- | --- | --- | --- | --- | --- | --- | --- |
| Accession | Gene names | MW [kDa] | Protein score | Sequence coverage (%) | # Unique Peptides | # Peptides | # PSMs |
| Q08345 | DDR1 | 101.06 | 1170.735 | 12.16 | 9 | 9 | 24 |
| P08779 | KRT16 | 51.24 | 709.43 | 21.78 | 1 | 10 | 29 |
| Q9BQE3 | TUBA1C | 49.86 | 475.79 | 14.48 | 5 | 5 | 15 |
| Q5D862 | FLG2 | 247.93 | 123.38 | 0.92 | 2 | 2 | 2 |
| P00367 | GLUD1 | 61.36 | 110.20 | 4.84 | 2 | 2 | 2 |
| Q03252 | LMNB2 | 69.91 | 106.34 | 3.55 | 1 | 2 | 2 |
| P01889 | HLA-B | 40.43 | 94.05 | 6.63 | 2 | 2 | 2 |
| P08195 | SLC3A2 | 67.95 | 90.94 | 2.06 | 1 | 1 | 1 |
| P02765 | AHSG | 39.30 | 89.02 | 5.18 | 2 | 2 | 2 |
| Q99729 | HNRNPAB | 36.20 | 81.77 | 6.93 | 1 | 2 | 3 |
| Q32MZ4 | LRRFIP1 | 89.20 | 78.72 | 1.73 | 1 | 1 | 1 |
| P30153 | PPP2R1A | 65.27 | 73.63 | 1.7 | 1 | 1 | 1 |
| Q9BXP5 | SRRT | 100.60 | 73.48 | 2.4 | 2 | 2 | 2 |
| Q96P70 | IPO9 | 115.89 | 71.97 | 2.02 | 1 | 1 | 1 |
| Q9Y2B0 | CNPY2 | 20.64 | 71.67 | 8.79 | 1 | 1 | 1 |
| Q8NEF9 | SRFBP1 | 48.60 | 70.18 | 3.26 | 1 | 1 | 1 |
| P33993 | MCM7 | 81.26 | 70.17 | 1.81 | 1 | 1 | 1 |
| Q13620 | CUL4B | 103.92 | 69.47 | 2.19 | 2 | 2 | 2 |
| P61604 | HSPE1 | 10.92 | 67.17 | 13.73 | 1 | 1 | 2 |
| P60228 | EIF3E | 52.19 | 66.61 | 3.6 | 2 | 2 | 2 |
| P42677 | RPS27 | 9.45 | 66.01 | 9.52 | 1 | 1 | 1 |
| Q13263 | TRIM28 | 88.49 | 64.96 | 3.59 | 1 | 1 | 1 |
| P16070 | CD44 | 81.49 | 64.48 | 1.08 | 1 | 1 | 1 |
| P31930 | UQCRC1 | 52.61 | 63.49 | 2.5 | 1 | 1 | 1 |
| Q08J23 | NSUN2 | 86.42 | 62.48 | 1.3 | 1 | 1 | 1 |
| P13637 | ATP1A3 | 111.68 | 61.96 | 1.48 | 1 | 1 | 1 |
| P14324 | FDPS | 48.24 | 60.02 | 4.3 | 2 | 2 | 2 |
| Q86U42 | PABPN1 | 32.73 | 59.90 | 5.23 | 1 | 1 | 1 |
| P36543 | ATP6V1E1 | 26.13 | 58.88 | 3.54 | 1 | 1 | 1 |
| Q86TI2 | DPP9 | 98.20 | 58.70 | 1.27 | 1 | 1 | 1 |
| Q96RS6 | NUDCD1 | 66.71 | 57.90 | 1.89 | 1 | 1 | 1 |
| P28070 | PSMB4 | 29.19 | 57.19 | 3.79 | 1 | 1 | 1 |
| Q9NX63 | CHCHD3 | 26.14 | 57.04 | 3.96 | 1 | 1 | 1 |
| Q15642 | TRIP10 | 68.31 | 56.27 | 1.33 | 1 | 1 | 1 |
| A6NEC2 | NPEPPSL1 | 53.71 | 56.14 | 1.88 | 1 | 1 | 1 |
| P51858 | HDGF | 26.77 | 52.83 | 8.75 | 2 | 2 | 2 |
| Q07960 | ARHGAP1 | 50.40 | 52.35 | 1.59 | 1 | 1 | 1 |
| Q04446 | GBE1 | 80.42 | 52.33 | 1.57 | 1 | 1 | 1 |
| A0A075B759 | PPIAL4E | 18.19 | 51.95 | 3.66 | 1 | 1 | 1 |
| Q8WWH5 | TRUB1 | 37.23 | 51.63 | 4.01 | 1 | 1 | 1 |
| P07305 | H1F0 | 20.85 | 51.56 | 5.15 | 1 | 1 | 1 |
| P63241 | EIF5A | 16.82 | 50.27 | 5.19 | 1 | 1 | 1 |
| O75964 | ATP5L | 11.42 | 49.50 | 10.68 | 1 | 1 | 1 |
| Q16401 | PSMD5 | 56.16 | 49.34 | 1.79 | 1 | 1 | 1 |
| O14980 | XPO1 | 123.31 | 49.25 | 0.93 | 1 | 1 | 1 |
| Q15758 | SLC1A5 | 56.56 | 49.00 | 1.66 | 1 | 1 | 1 |
| P0CG48 | UBC | 76.99 | 46.37 | 21.02 | 1 | 1 | 1 |
| O15427 | SLC16A3 | 49.44 | 45.28 | 2.8 | 1 | 1 | 1 |
| Q9UL46 | PSME2 | 27.38 | 43.60 | 3.77 | 1 | 1 | 1 |
| Q14566 | MCM6 | 92.83 | 43.58 | 1.22 | 1 | 1 | 1 |
| P52294 | KPNA1 | 60.18 | 43.38 | 2.04 | 1 | 1 | 1 |
| O00505 | KPNA3 | 57.77 | 43.18 | 1.34 | 1 | 1 | 1 |
| P01699 | IGLV1-44 | 12.19 | 42.70 | 5.98 | 1 | 1 | 1 |
| Q5XPI4 | RNF123 | 148.42 | 42.16 | 0.46 | 1 | 1 | 1 |
| Q9UNH7 | SNX6 | 46.62 | 41.94 | 1.97 | 1 | 1 | 1 |
| P62633 | CNBP | 19.45 | 41.34 | 4.52 | 1 | 1 | 1 |
| Q6NUK1 | SLC25A24 | 53.32 | 41.19 | 1.89 | 1 | 1 | 1 |
| P31949 | S100A11 | 11.73 | 40.36 | 8.57 | 1 | 1 | 1 |
| P05121 | SERPINE1 | 45.03 | 40.11 | 2.49 | 1 | 1 | 1 |
| Q9H1E3 | NUCKS1 | 27.28 | 39.66 | 3.7 | 1 | 1 | 1 |
| Q16658 | FSCN1 | 54.50 | 39.25 | 1.83 | 1 | 1 | 1 |
| O75083 | WDR1 | 66.15 | 39.21 | 1.32 | 1 | 1 | 1 |
| P62330 | ARF6 | 20.07 | 38.95 | 3.43 | 1 | 1 | 1 |
| Q9Y241 | HIGD1A | 10.14 | 38.89 | 19.35 | 1 | 1 | 1 |
| Q9Y696 | CLIC4 | 28.75 | 38.44 | 4.74 | 1 | 1 | 1 |
| Q02790 | FKBP4 | 51.77 | 38.32 | 1.53 | 1 | 1 | 1 |
| Q9NZT1 | CALML5 | 15.88 | 38.00 | 8.9 | 1 | 1 | 1 |
| P51153 | RAB13 | 22.76 | 36.13 | 4.43 | 1 | 1 | 1 |
| Q13098 | GPS1 | 55.50 | 35.95 | 3.05 | 1 | 1 | 1 |
| P24534 | EEF1B2 | 24.75 | 35.80 | 3.11 | 1 | 1 | 1 |
| P00492 | HPRT1 | 24.56 | 35.61 | 5.5 | 1 | 1 | 1 |
| P63173 | RPL38 | 8.21 | 33.58 | 14.29 | 1 | 1 | 1 |
| Q14790 | CASP8 | 55.36 | 33.06 | 2.3 | 1 | 1 | 1 |
| Q14011 | CIRBP | 18.64 | 30.94 | 6.4 | 1 | 1 | 1 |

| **Supplementary Table S6. Proteins bind with DDR1 identified by IP-MS in HLF cells.** | | | | | | | |
| --- | --- | --- | --- | --- | --- | --- | --- |
| Accession | Gene names | MW [kDa] | Protein score | Sequence coverage (%) | # Unique Peptides | # Peptides | # PSMs |
| Q08345 | DDR1 | 101.063 | 903.0282 | 29.03 | 20 | 20 | 25 |
| P68032 | ACTC1 | 41.99 | 777.97 | 26.53 | 1 | 10 | 20 |
| P68363 | TUBA1B | 50.12 | 769.84 | 40.13 | 0 | 13 | 18 |
| Q13310 | PABPC4 | 70.74 | 219.53 | 9.16 | 1 | 4 | 4 |
| O00425 | IGF2BP3 | 63.67 | 167.32 | 6.91 | 2 | 3 | 3 |
| Q96I59 | NARS2 | 54.06 | 167.24 | 9.01 | 4 | 4 | 4 |
| Q04695 | KRT17 | 48.08 | 162.33 | 12.27 | 1 | 5 | 5 |
| P22392 | NME2 | 17.29 | 160.40 | 34.87 | 1 | 4 | 6 |
| P29966 | MARCKS | 31.54 | 159.05 | 20.78 | 3 | 3 | 3 |
| Q9UNX3 | RPL26L1 | 17.25 | 158.48 | 37.24 | 1 | 7 | 7 |
| Q9Y678 | COPG1 | 97.66 | 150.16 | 6.75 | 4 | 4 | 4 |
| P80723 | BASP1 | 22.68 | 150.09 | 45.81 | 5 | 5 | 5 |
| Q10567 | AP1B1 | 104.57 | 120.13 | 3.79 | 1 | 2 | 2 |
| P60953 | CDC42 | 21.25 | 118.56 | 8.9 | 1 | 1 | 2 |
| P08574 | CYC1 | 35.40 | 112.97 | 6.77 | 2 | 2 | 3 |
| Q9Y305 | ACOT9 | 49.87 | 108.15 | 7.52 | 2 | 2 | 2 |
| P22087 | FBL | 33.76 | 105.23 | 10.59 | 3 | 3 | 3 |
| Q96S44 | TP53RK | 28.14 | 102.46 | 11.07 | 2 | 2 | 2 |
| P42285 | SKIV2L2 | 117.73 | 102.35 | 2.21 | 1 | 1 | 2 |
| Q96QV6 | HIST1H2AA | 14.22 | 97.18 | 22.9 | 1 | 2 | 4 |
| O76094 | SRP72 | 74.56 | 94.91 | 4.47 | 2 | 2 | 2 |
| Q96AG4 | LRRC59 | 34.91 | 85.83 | 6.84 | 2 | 2 | 2 |
| Q13126 | MTAP | 31.22 | 83.45 | 12.37 | 2 | 2 | 2 |
| O14556 | GAPDHS | 44.47 | 83.13 | 4.41 | 1 | 2 | 3 |
| Q92979 | EMG1 | 26.70 | 81.20 | 12.7 | 2 | 2 | 2 |
| Q9BT67 | NDFIP1 | 24.88 | 80.08 | 7.69 | 1 | 1 | 1 |
| O60832 | DKC1 | 57.64 | 79.54 | 6.42 | 2 | 2 | 2 |
| P15121 | AKR1B1 | 35.83 | 78.56 | 5.06 | 2 | 2 | 2 |
| Q01844 | EWSR1 | 68.44 | 76.30 | 2.9 | 1 | 1 | 1 |
| P30153 | PPP2R1A | 65.27 | 75.88 | 3.06 | 1 | 1 | 1 |
| Q96AE4 | FUBP1 | 67.52 | 75.66 | 3.57 | 2 | 2 | 2 |
| Q92973 | TNPO1 | 102.29 | 75.54 | 1.34 | 1 | 1 | 1 |
| P55145 | MANF | 20.69 | 74.74 | 25.27 | 3 | 3 | 3 |
| P28838 | LAP3 | 56.13 | 74.73 | 5.01 | 2 | 2 | 2 |
| O15479 | MAGEB2 | 35.26 | 74.42 | 5.02 | 1 | 1 | 1 |
| Q15785 | TOMM34 | 34.54 | 74.16 | 4.85 | 1 | 1 | 1 |
| Q9BY44 | EIF2A | 64.95 | 71.72 | 3.93 | 2 | 2 | 2 |
| Q12904 | AIMP1 | 34.33 | 71.57 | 10.58 | 2 | 2 | 2 |
| P62633 | CNBP | 19.45 | 71.05 | 12.99 | 2 | 2 | 2 |
| P30040 | ERP29 | 28.98 | 70.25 | 7.66 | 2 | 2 | 2 |
| Q01970 | PLCB3 | 138.71 | 69.77 | 0.97 | 1 | 1 | 1 |
| P61006 | RAB8A | 23.65 | 69.42 | 12.08 | 1 | 2 | 2 |
| P24539 | ATP5F1 | 28.89 | 68.61 | 4.69 | 1 | 1 | 1 |
| Q9HC35 | EML4 | 108.85 | 68.29 | 2.85 | 2 | 2 | 2 |
| Q99439 | CNN2 | 33.68 | 67.95 | 5.5 | 1 | 1 | 1 |
| P11233 | RALA | 23.55 | 64.99 | 6.8 | 1 | 1 | 1 |
| P54578 | USP14 | 56.03 | 64.85 | 2.63 | 1 | 1 | 1 |
| P14550 | AKR1A1 | 36.55 | 64.33 | 9.54 | 2 | 2 | 2 |
| O00764 | PDXK | 35.08 | 64.11 | 6.73 | 1 | 1 | 1 |
| O75251 | NDUFS7 | 23.55 | 63.71 | 4.23 | 1 | 1 | 1 |
| Q8WXF1 | PSPC1 | 58.71 | 62.80 | 4.78 | 2 | 2 | 2 |
| Q15050 | RRS1 | 41.17 | 62.68 | 4.11 | 2 | 2 | 2 |
| Q96QR8 | PURB | 33.22 | 62.40 | 11.86 | 2 | 2 | 2 |
| Q96EP5 | DAZAP1 | 43.36 | 62.01 | 3.93 | 1 | 1 | 1 |
| Q9NWH9 | SLTM | 117.08 | 61.75 | 1.84 | 1 | 1 | 1 |
| P49755 | TMED10 | 24.96 | 61.19 | 5.48 | 1 | 1 | 1 |
| Q86U42 | PABPN1 | 32.73 | 60.62 | 3.59 | 1 | 1 | 1 |
| Q15758 | SLC1A5 | 56.56 | 58.14 | 2.03 | 1 | 1 | 1 |
| Q92945 | KHSRP | 73.07 | 56.65 | 1.55 | 1 | 1 | 1 |
| P23193 | TCEA1 | 33.95 | 56.21 | 6.64 | 2 | 2 | 2 |
| Q71RC2 | LARP4 | 80.55 | 55.18 | 1.8 | 1 | 1 | 1 |
| P00568 | AK1 | 21.62 | 54.80 | 6.19 | 1 | 1 | 1 |
| Q16795 | NDUFA9 | 42.48 | 54.65 | 3.45 | 1 | 1 | 1 |
| P11172 | UMPS | 52.19 | 53.03 | 2.08 | 1 | 1 | 1 |
| Q9BSD7 | NTPCR | 20.70 | 52.96 | 7.89 | 1 | 1 | 1 |
| Q15631 | TSN | 26.17 | 52.42 | 5.7 | 1 | 1 | 1 |
| P52701 | MSH6 | 152.69 | 50.78 | 0.88 | 1 | 1 | 1 |
| O75569 | PRKRA | 34.38 | 50.28 | 6.07 | 1 | 1 | 1 |
| P42765 | ACAA2 | 41.90 | 50.05 | 2.52 | 1 | 1 | 1 |
| Q8WVC0 | LEO1 | 75.36 | 49.48 | 1.95 | 1 | 1 | 1 |
| Q86TB9 | PATL1 | 86.80 | 49.40 | 1.82 | 1 | 1 | 1 |
| O43670 | ZNF207 | 50.72 | 48.95 | 2.72 | 1 | 1 | 1 |
| Q9H5Q4 | TFB2M | 45.32 | 48.38 | 4.04 | 1 | 1 | 1 |
| Q00688 | FKBP3 | 25.16 | 48.36 | 4.91 | 1 | 1 | 1 |
| P04792 | HSPB1 | 22.7685 | 48.34 | 4.88 | 1 | 1 | 1 |
| Q9GZT3 | SLIRP | 12.3414 | 47.74 | 12.84 | 1 | 1 | 1 |
| Q32MZ4 | LRRFIP1 | 89.1987 | 47.59 | 1.24 | 1 | 1 | 1 |
| Q7L1Q6 | BZW1 | 48.0127 | 47.49 | 6.21 | 1 | 1 | 1 |
| P57081 | WDR4 | 45.461 | 47.43 | 2.67 | 1 | 1 | 1 |
| P20742 | PZP | 163.76 | 46.38 | 0.81 | 1 | 1 | 1 |
| Q86U38 | NOP9 | 69.3945 | 46.32 | 2.2 | 1 | 1 | 1 |
| O95456 | PSMG1 | 32.8325 | 45.87 | 3.47 | 1 | 1 | 1 |
| Q96P11 | NSUN5 | 46.6623 | 45.67 | 3.73 | 1 | 1 | 1 |
| Q96Q11 | TRNT1 | 50.0961 | 45.12 | 3.46 | 1 | 1 | 1 |
| O95166 | GABARAP | 13.9093 | 44.81 | 5.98 | 1 | 1 | 1 |
| Q9UNZ5 | C19orf53 | 10.5701 | 44.55 | 9.09 | 1 | 1 | 1 |
| Q92541 | RTF1 | 80.2646 | 43.78 | 1.69 | 1 | 1 | 1 |
| Q9P035 | HACD3 | 43.1316 | 43.74 | 3.04 | 1 | 1 | 1 |
| P09543 | CNP | 47.5487 | 43.73 | 5.46 | 1 | 1 | 1 |
| P28072 | PSMB6 | 25.3414 | 43.53 | 4.6 | 1 | 1 | 1 |
| Q96KB5 | PBK | 36.062 | 43.37 | 2.8 | 1 | 1 | 1 |
| Q13895 | BYSL | 49.57 | 43.02 | 2.06 | 1 | 1 | 1 |
| P42330 | AKR1C3 | 36.8298 | 42.64 | 2.48 | 1 | 1 | 1 |
| P25788 | PSMA3 | 28.4151 | 42.05 | 3.92 | 1 | 1 | 1 |
| Q9NWX6 | THG1L | 34.8084 | 41.89 | 5.7 | 1 | 1 | 1 |
| P14324 | FDPS | 48.2446 | 41.7 | 2.86 | 1 | 1 | 1 |
| Q5SNV9 | C1orf167 | 162.321 | 41.29 | 0.54 | 1 | 1 | 1 |
| P53582 | METAP1 | 43.1873 | 41.22 | 5.18 | 1 | 1 | 1 |
| Q99575 | POP1 | 114.636 | 40.97 | 0.78 | 1 | 1 | 1 |
| Q9NZL9 | MAT2B | 37.5282 | 40.89 | 3.59 | 1 | 1 | 1 |
| P49458 | SRP9 | 10.1051 | 40.51 | 12.79 | 1 | 1 | 1 |
| Q9Y3D3 | MRPS16 | 15.335 | 40.38 | 5.84 | 1 | 1 | 1 |
| Q9BV44 | THUMPD3 | 56.9668 | 40.36 | 2.96 | 1 | 1 | 1 |
| P53990 | IST1 | 39.7255 | 40.35 | 5.22 | 1 | 1 | 1 |
| P30419 | NMT1 | 56.7699 | 40.32 | 1.41 | 1 | 1 | 1 |
| O14908 | GIPC1 | 36.0267 | 40.15 | 3.6 | 1 | 1 | 1 |
| Q5T2N8 | ATAD3C | 46.3501 | 40.15 | 1.95 | 1 | 1 | 1 |
| Q9UHD1 | CHORDC1 | 37.4656 | 40.07 | 5.42 | 1 | 1 | 1 |
| O95232 | LUC7L3 | 51.4351 | 39.71 | 3.7 | 1 | 1 | 1 |
| Q9ULX3 | NOB1 | 46.6458 | 39.57 | 2.18 | 1 | 1 | 1 |
| Q01581 | HMGCS1 | 57.2574 | 39.54 | 2.5 | 1 | 1 | 1 |
| Q14847 | LASP1 | 29.6982 | 39.24 | 2.68 | 1 | 1 | 1 |
| O43847 | NRDC | 131.488 | 38.76 | 0.96 | 1 | 1 | 1 |
| Q7L0Y3 | TRMT10C | 47.3166 | 38.55 | 2.23 | 1 | 1 | 1 |
| A0FGR8 | ESYT2 | 102.294 | 38.49 | 1.3 | 1 | 1 | 1 |
| P43246 | MSH2 | 104.677 | 38.47 | 1.5 | 1 | 1 | 1 |
| Q9H814 | PHAX | 44.3751 | 38.39 | 2.79 | 1 | 1 | 1 |
| Q96KP4 | CNDP2 | 52.8449 | 38.35 | 4.42 | 1 | 1 | 1 |
| O15145 | ARPC3 | 20.5334 | 38.18 | 7.3 | 1 | 1 | 1 |
| Q9BUT1 | BDH2 | 26.7067 | 38.17 | 4.9 | 1 | 1 | 1 |
| P06493 | CDK1 | 34.0739 | 38.11 | 2.36 | 1 | 1 | 1 |
| Q13155 | AIMP2 | 35.3263 | 38.02 | 3.75 | 1 | 1 | 1 |
| Q9UNM6 | PSMD13 | 42.9181 | 37.8 | 2.66 | 1 | 1 | 1 |
| P40261 | NNMT | 29.5551 | 37.65 | 6.44 | 1 | 1 | 1 |
| Q15208 | STK38 | 54.1554 | 37.43 | 2.15 | 1 | 1 | 1 |
| P49588 | AARS | 106.743 | 37.41 | 1.24 | 1 | 1 | 1 |
| Q9BQ75 | CMSS1 | 31.864 | 37.35 | 2.15 | 1 | 1 | 1 |
| P51572 | BCAP31 | 27.974 | 37.02 | 3.25 | 1 | 1 | 1 |
| P61011 | SRP54 | 55.6683 | 37.01 | 1.39 | 1 | 1 | 1 |
| P54819 | AK2 | 26.4608 | 36.83 | 5.44 | 1 | 1 | 1 |
| Q15029 | EFTUD2 | 109.366 | 36.28 | 1.65 | 1 | 1 | 1 |
| P05067 | APP | 86.8881 | 36.23 | 1.56 | 1 | 1 | 1 |
| O43148 | RNMT | 54.8093 | 36.06 | 2.1 | 1 | 1 | 1 |
| Q9BYD3 | MRPL4 | 34.8973 | 35.91 | 5.14 | 1 | 1 | 1 |
| P30405 | PPIF | 22.0262 | 35.79 | 3.86 | 1 | 1 | 1 |
| Q96PK6 | RBM14 | 69.4489 | 35.43 | 2.24 | 1 | 1 | 1 |
| P61326 | MAGOH | 17.1528 | 35.4 | 6.85 | 1 | 1 | 1 |
| Q9BZE4 | GTPBP4 | 73.9178 | 35.39 | 1.89 | 1 | 1 | 1 |
| P33991 | MCM4 | 96.4976 | 35.28 | 1.27 | 1 | 1 | 1 |
| P60981 | DSTN | 18.4935 | 35.22 | 7.27 | 1 | 1 | 1 |
| Q15392 | DHCR24 | 60.062 | 35.2 | 1.74 | 1 | 1 | 1 |
| O14602 | EIF1AY | 16.4323 | 35.17 | 7.64 | 1 | 1 | 1 |
| P19823 | ITIH2 | 106.397 | 35.16 | 1.59 | 1 | 1 | 1 |
| Q9NRP0 | OSTC | 16.8178 | 35.15 | 8.05 | 1 | 1 | 1 |
| P13798 | APEH | 81.1726 | 35.1 | 1.64 | 1 | 1 | 1 |
| Q13620 | CUL4B | 103.916 | 34.76 | 1.31 | 1 | 1 | 1 |
| O00193 | SMAP | 20.3202 | 34.58 | 3.83 | 1 | 1 | 1 |
| Q9UL42 | PNMA2 | 41.4832 | 34.55 | 2.75 | 1 | 1 | 1 |
| Q96PZ0 | PUS7 | 74.9882 | 34.18 | 1.66 | 1 | 1 | 1 |
| O95407 | TNFRSF6B | 32.6583 | 33.97 | 2 | 1 | 1 | 1 |
| P62891 | RPL39 | 6.40265 | 33.74 | 19.61 | 1 | 1 | 1 |
| P20339 | RAB5A | 23.6438 | 33.64 | 5.12 | 1 | 1 | 1 |
| P0DMM9 | SULT1A3 | 34.1741 | 33.31 | 2.37 | 1 | 1 | 1 |
| O95218 | ZRANB2 | 37.382 | 33.11 | 2.73 | 1 | 1 | 1 |
| Q8IWS0 | PHF6 | 41.264 | 32.74 | 2.47 | 1 | 1 | 1 |
| Q05682 | CALD1 | 93.1754 | 31.75 | 1.39 | 1 | 1 | 1 |
| Q8NEF9 | SRFBP1 | 48.6044 | 31.43 | 1.86 | 1 | 1 | 1 |
| Q8IW75 | SERPINA12 | 47.145 | 31.04 | 1.93 | 1 | 1 | 1 |
| P62330 | ARF6 | 20.0694 | 30.33 | 3.43 | 1 | 1 | 1 |
| Q9H492 | MAP1LC3A | 14.2634 | 30.18 | 5.79 | 1 | 1 | 1 |
| Q9P0V9 | SEPT10 | 52.5596 | 25.32 | 1.98 | 1 | 1 | 1 |

**Supplementary Table S7. Correlation between PSD4 Expression with Clinicopathologic Features in HCC (n=169)**

| **Clinicopathological Features** | **Total** | **Relative PSD4 Expression** | | ***P* value** |
| --- | --- | --- | --- | --- |
|  |  | **Low** | **High** |  |
| ***Sex*** |  |  |  |  |
| Male | 145 | 80 | 65 | 0.111 |
| Female | 24 | 17 | 7 |  |
| ***Age(years)*** |  |  |  |  |
| ≤50 | 87 | 46 | 41 | 0.142 |
| ＞50 | 82 | 51 | 31 |  |
| ***ALT(ng/ml)*** |  |  |  |  |
| ≤75 | 137 | 81 | 56 | 0.229 |
| ＞75 | 32 | 16 | 16 |  |
| ***Serum AFP (ng/ml)*** |  |  |  |  |
| ＜400 | 92 | 52 | 40 | 0.462 |
| ≥400 | 77 | 45 | 32 |  |
| ***GGT(u/l)*** |  |  |  |  |
| ≤54 | 68 | 43 | 25 | 0.135 |
| ＞54 | 101 | 54 | 47 |  |
| ***HBV*** |  |  |  |  |
| Negative | 21 | 11 | 10 | 0.394 |
| Positive | 148 | 86 | 62 |  |
| ***HCV*** |  |  |  |  |
| Negative | 167 | 97 | 70 | 0.180* |
| Positive | 2 | 0 | 2 |  |
| ***Differentiation*** |  |  |  |  |
| Well/moderate | 88 | 57 | 31 | **0.031** |
| Poor | 81 | 40 | 41 |  |
| ***Tumor size(cm)^#^*** |  |  |  |  |
| ＜5 | 75 | 44 | 31 | 0.444 |
| ≥5 | 94 | 53 | 41 |  |
| ***Tumor encapsulation*** |  |  |  |  |
| Present | 74 | 53 | 21 | **0.001** |
| Absent | 95 | 44 | 51 |  |
| ***Tumor number*** |  |  |  |  |
| Single | 133 | 71 | 62 | 0.057 |
| Multiple | 36 | 26 | 10 |  |
| ***TNM stage*** |  |  |  |  |
| I | 91 | 65 | 26 | **0.001** |
| II+III+IV | 78 | 32 | 46 |  |
| ***Recurrence*** |  |  |  |  |
| Yes | 89 | 34 | 55 | **0.001** |
| No | 80 | 63 | 17 |  |

NOTE. Statistical significance (P <0.05) is shown in bold

^#^ Tumor size was measured by the length of the largest tumor nodule.

*; Fisher exact test.

| **Supplementary Table S8. Antibodies used in this study.** | | |
| --- | --- | --- |
| Antigens | Manufacturers | Application |
| GAPDH | KC-5G4, Kang Chen Bio-tech, Shanghai, China | 1:10000 for WB |
| anti-rabbit IgG HRP conjugated | Jackson ImmunoResearch Laboratories, Inc. West Grove, PA, USA | 1:5000 for WB |
| anti- mouse IgG HRP conjugated | Jackson ImmunoResearch Laboratories, Inc. West Grove, PA, USA | 1:5000 for WB |
| DDR1 | #5583, Cell Signaling Technology, Beverly, MA, USA | 1:1000 for WB; 1:300 for IF; 1:100 for IP |
| DDR1  p-DDR1 (Y513） | sc-532, Santa Cruz, CA, USA  #14531, Cell Signaling Technology, Beverly, MA, USA | 1:50 for IHC  1:1000 for WB |
| ERK | #4695, Cell Signaling Technology, Beverly, MA, USA | 1:2000 for WB |
| p-ERK (T202/Y204） | #4370, Cell Signaling Technology, Beverly, MA, USA | 1:1000 for WB |
| HA | #3724, Cell Signaling Technology, Beverly, MA, USA | 1:2000 for WB; 1:800 for IF; 1:50 for IP |
| FLAG | F1804, Sigma-Aldrich, St. Louis, MO, USA | 1:2000 for WB; 1:400 for IF; 1:500 for IP |
| MYC-Tag | #2276, Cell Signaling Technology, Beverly, MA, USA | 1:2000 for WB; 1:400 for IF; 1:500 for IP |
| p38 | #8690, Cell Signaling Technology, Beverly, MA, USA | 1:2000 for WB |
| p-p38 (T180/Y182) | #4511, Cell Signaling Technology, Beverly, MA, USA | 1:1000 for WB |
| c-Jun | #9165, Cell Signaling Technology, Beverly, MA, USA | 1:2000 for WB |
| p-c-jun (Ser73) | #3270, Cell Signaling Technology, Beverly, MA, USA | 1:1000 for WB |
| p-Tyrosine | 05-321, Millipore, CA, USA | 1:1000 for WB |
| ARF6 | #5740, Cell Signaling Technology, Beverly, MA, USA | 1:1000 for WB; 1:50 for IP |
| ARF6 | sc-7971, Santa Cruz, CA, USA | 1:50 for IF |
| PSD4 | ab154008, Abcam, Cambridge, England | 1:1000 for WB |
| PSD4 | SAB2700678, Sigma-Aldrich, St. Louis, MO, USA | 1:100 for IHC |
| IHC, immunohistochemistry; IF, immunofluorescence; WB, Western Blot; IP, immunoprecipitation. | | |

**Supplementary Table S9. The sequences of shRNA oligo pairs.**

| **Identifier** | **Sequence (5’ to 3’)** |
| --- | --- |
| DDR1-shRNA#2 sense | CCGGACTCCACCTATGACGGACATACTCGAGTATGTCCGTCATAGGTGGAGTTTTTTG |
| DDR1-shRNA#2 antisense | AATTCAAAAAACTCCACCTATGACGGACATACTCGAGTATGTCCGTCATAGGTGGAGT |
|  |  |
| DDR1-shRNA#3 sense | CCGGCCTATACGTTTCTGTGGAGTACTCGAGTACTCCACAGAAACGTATAGGTTTTTG |
| DDR1-shRNA#3 antisense | AATTCAAAAACCTATACGTTTCTGTGGAGTACTCGAGTACTCCACAGAAACGTATAGG |
|  |  |
|  |  |
| ARF6-shRNA#1 sense | CCGGCAACAATCCTGTACAAGTTGACTCGAGTCAACTTGTACAGGATTGTTGTTTTTG |
| ARF6-shRNA#1 antisense | AATTCAAAAACAACAATCCTGTACAAGTTGACTCGAGTCAACTTGTACAGGATTGTTG |
|  |  |
| ARF6-shRNA#2 sense | CCGGAGCTGCACCGCATTATCAATGCTCGAGCATTGATAATGCGGTGCAGCTTTTTTTG |
| ARF6-shRNA#2 antisense | AATTCAAAAAAAGCTGCACCGCATTATCAATGCTCGAGCATTGATAATGCGGTGCAGCT |
|  |  |
|  |  |
| PSD4-shRNA#1 sense | CCGGTCCCTGGAGGAGACTTATTTCCTCGAGGAAATAAGTCTCCTCCAGGGATTTTTTG |
| PSD4-shRNA#1 antisense | AATTCAAAAAATCCCTGGAGGAGACTTATTTCCTCGAGGAAATAAGTCTCCTCCAGGGA |
|  |  |
| PSD4-shRNA#2 sense | CCGGTTCCCTGTGCCCATCTATAAACTCGAGTTTATAGATGGGCACAGGGAATTTTTTG |
| PSD4-shRNA#2 antisense | AATTCAAAAAATTCCCTGTGCCCATCTATAAACTCGAGTTTATAGATGGGCACAGGGAA |
|  |  |
| PSD4-shRNA#3 sense | CCGGTGCAGAAGAACAATGACTTTACTCGAGTAAAGTCATTGTTCTTCTGCATTTTTTG |
| PSD4-shRNA#3 antisense | AATTCAAAAAATGCAGAAGAACAATGACTTTACTCGAGTAAAGTCATTGTTCTTCTGCA |

**Supplementary Table S10. Sequences of gene-specific primers used for qRT-PCR.**

| Gene | Forward (5’-3’) | Reverse (5’-3’) |
| --- | --- | --- |
| DDR1 | CCGACTGGTTCGCTTCTACC | CGGTGTAAGACAGGAGTCCATC |
| ARF6 | GGGAAGGTGCTATCCAAAATCTT | CACATCCCATACGTTGAACTTGA |
| PSD4 | TCAACCTGTACTTGGGAGACA | GCCCCAGGGAGGAACATTTT |
| GAPDH | GGAGCGAGATCCCTCCAAAAT | GGCTGTTGTCATACTTCTCATGG |
